# Ginsenoside Rh2 sensitizes the anti-cancer effects of sunitinib by inducing cell cycle arrest in renal cell carcinoma

**DOI:** 10.1038/s41598-022-20075-0

**Published:** 2022-11-17

**Authors:** Hyun Ji Hwang, Seong Hwi Hong, Hong Sang Moon, Young Eun Yoon, Sung Yul Park

**Affiliations:** 1 Department of Urology, Hanyang University College of Medicine, Seoul, 04763 South Korea; 2 Department of Translational Medicine, Hanyang University Graduate School of Biomedical Science & Engineering, Seoul, 04763 South Korea

**Keywords:** Cancer, Cell biology, Molecular biology, Oncology

## Abstract

Sunitinib, a VEGF blockade, is used to treat clear cell renal cell carcinoma (ccRCC). However, the anti-cancer treatment effects of sunitinib do not last long in ccRCC patients. Ginsenoside, a natural medicine extracted from ginseng, has been studied in cancer treatment and shown to have anti-tumor effects and low toxicity. We assessed cell viability and cell cycle analysis in ccRCC cell lines after treatment with ginsenoside and sunitinib. DNA damage was evaluated by measuring 8-OHdG levels and comet assay. ROS levels, reflecting the cause of oxidative stress, were also measured. Ginsenoside significantly enhanced the inhibition of cell viability by sunitinib, a result that was also confirmed in the xenograft model. In cell cycle analysis, combination treatment of ginsenoside and sunitinib enhanced G2M arrest in comparison with single-treatment groups. In addition, DNA damage was increased by ginsenoside and sunitinib according to the comet assay, and the level of 8-OHdG, which reflects oxidative DNA damage, also increased. We verified that ginsenoside enhances the efficacy of sunitinib to inhibit the proliferation of ccRCC cells via induction of oxidative DNA damage. The combination therapy of sunitinib and ginsenoside suggested the possibility of effectively treating ccRCC patients.

## Introduction

Renal cell carcinoma (RCC) is the most common type of kidney cancer. Among RCCs, clear cell RCC (ccRCC) accounts for approximately 75% of RCC^1^. In ccRCC, von Hippel-Lindau (VHL) disease results from a major genetic mutation and is characterized by an E3 ubiquitin ligase that induces degradation of hypoxia inducing factor-alpha (HIF alpha) under normoxic conditions^2^. Genetic VHL inactivation of ccRCC causes constitutive HIF alpha accumulation and consequent upregulation of hypoxia-related genes^3–8^. Histologically, ccRCC is a hyper-vascular tumor caused by the upregulation of angiogenesis-associated genes such as vascular endothelial growth factor (VEGF), a downstream product of the hypoxia pathway^9,10^. VEGF binds the vascular endothelial growth factor receptor (VEGFR) which is the tyrosine kinase receptor (TKR), to induce angiogenesis^11^.

Anti-angiogenic drugs are used sequentially to prolong clinical benefit in patients with recurrent disease. Among anti-angiogenic drugs inhibiting VEGF/VEGFR pathways, sunitinib, a tyrosine kinase receptor inhibitor (TKI), is the classic first-line drug for RCC^12^. However, 10–20% of advanced RCC patients are intrinsically refractory to sunitinib, and the remaining patients typically experience tumor progression after 6–15 months of therapy, meaning sunitinib fails to successfully prolong the survival of RCC patients^13^. Because RCC is difficult to completely treat with sunitinib, an alternative therapy is needed to increase the sensitivity of treatment and prevent the development of resistance to sunitinib in RCC.

The use of natural products for cancer treatment has been increasing. Effective treatment with lower toxicity compared to other drugs may be achieved by using natural products. Ginsenoside, a natural material derived from ginseng, has been studied in cancer treatment due to its anti-tumor effects and low toxicity^14^. There are more than 100 types of ginsenosides extracted from ginseng, including Rg3, Rh2, Rb1, and Rb2^15^. Rg3 and Rh2 are known to represent anti-cancer effects in various cancers including lung cancer, breast cancer, and prostate cancer by inducing inhibition of proliferation and invasion and DNA damage^16–20^. However, not much is known about the effects of Rg3 and Rh2 on ccRCC. In this study, we investigated the effects of Rg3 and Rh2 on RCC and whether co-treatment with ginsenoside and sunitinib work in ccRCC and explored the underlying mechanisms.

## Results

### Ginsenoside Rh2 enhanced the anti-cancer effect of sunitinib in ccRCC

**Figure 1:**
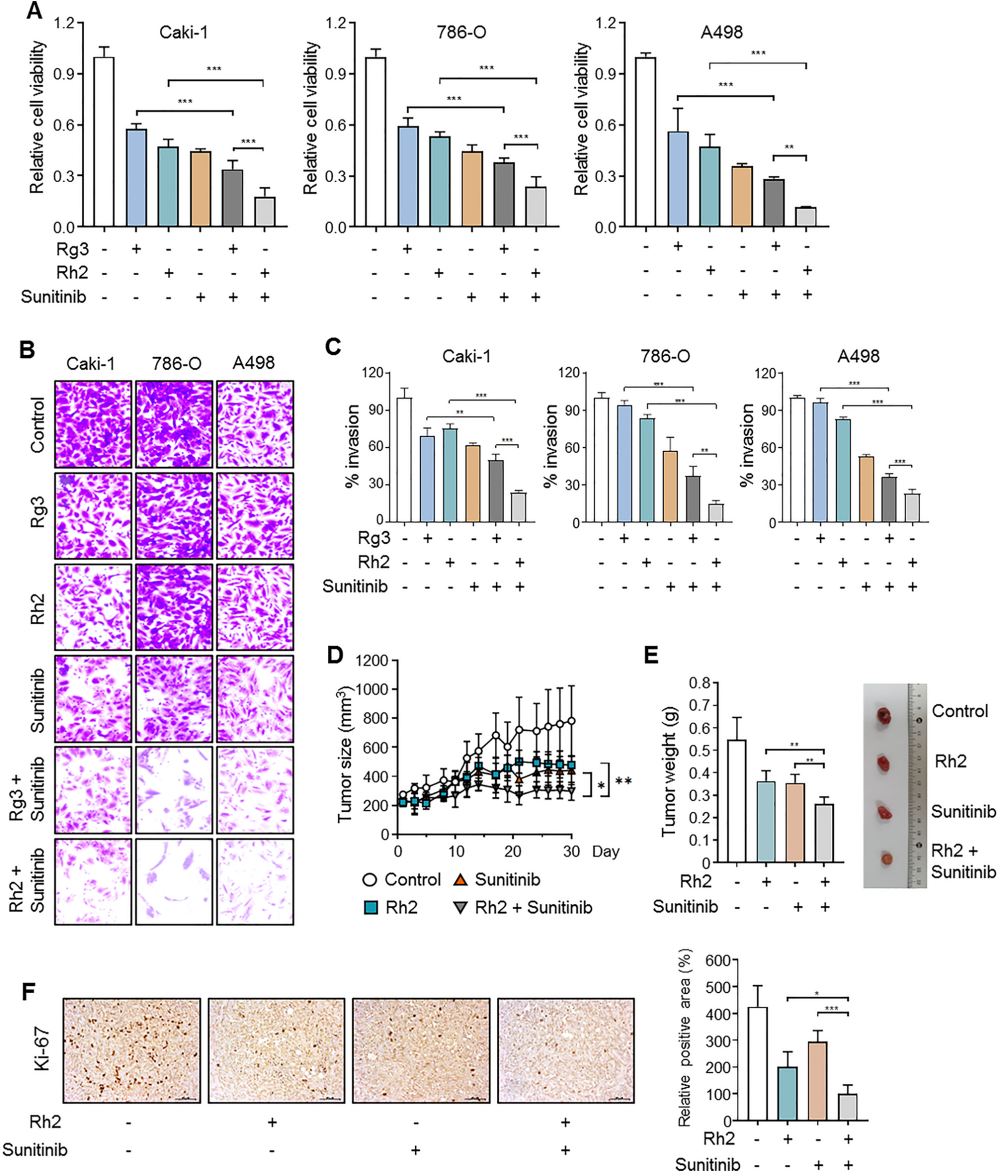
Ginsenoside enhances the anti-proliferative effects of sunitinib in ccRCC cell lines and the xenograft model. (**A**) Cell viability was measured after 24 h of drug co-treatment with ginsenoside and sunitinib. Caki-1, 786-O, and A498 cells were treated with Rh2 (10 μM), Rg3 (10 μM), and sunitinib (10 μM) (**p < 0.01, ***p < 0.001). Perform at least three biological repeats (n = 3). (**B**) The Matrigel invasion assay showed that anti-invasiveness significantly decreased in ccRCC cell lines after co-treatment with ginsenoside and sunitinib. The representative images were obtained after 24 h of drug treatment. (n = 3). (**C**) Numbers of invading cells in each group (**p < 0.01, ***p < 0.001). (n = 3). (**D**,**E**) A498 cells were injected subcutaneously into balb/c nude mice (n = 5). We evaluated tumor volumes and sizes in four treatment groups: DMSO, Rh2 (10 mg/kg), sunitinib (10 mg/kg), and Rh2 + sunitinib. Mice received intraperitoneal injections of ginsenoside three times per week and sunitinib was given by oral administration every day. All groups were evaluated three times per week. The tumor weights were evaluated after 4 weeks of treatment (*p < 0.05, **p < 0.01). (**F**) Immunohistochemistry (IHC) staining for Ki-67 in A498 xenograft treated with Rh2/sunitinib. We quantified the Ki-67 positive areas identified in five mice tissues per group (*p < 0.05, ***p < 0.001). (200 × magnification).

First, to evaluate whether ginsenoside Rg3 and Rh2 exhibit anti-cancer effects in renal cancer, we measured cell viability in three ccRCC cell lines (Caki-1, 786-O, and A498). Both ginsenoside Rg3 and Rh2 reduced ccRCC cell growth in a dose-dependent manner (Fig. S1A,B). Next, we measured cell viability to determine the most suitable concentration of sunitinib for measuring synergic effects with ginsenoside in ccRCC (Fig. S1C). We conducted combination treatment experiments using Rg3 10 μM, Rh2 10 μM, and sunitinib 10 μM, concentrations that reduced cell viability by about 50% in all three cell lines (Fig. S1A-C). Combination treatment with ginsenoside and sunitinib effectively reduced cell viability (Fig. 1A). To assess metastatic ability, we performed invasion assays using matrigel. Compared to single treatment, invasion ability was more effectively inhibited by combination treatment (Fig. 1B,C). Ginsenoside Rg3 and Rh2 both showed more effective anti-cancer effects when administered with sunitinib compared to single treatment. However, cell viability measured for the combination of ginsenoside and sunitinib indicates that Rh2 (Caki-1; 17.8%/ 786-O; 23.7%/ A498; 11.5%) is superior to Rg3 (Caki-1; 33.7%/ 786-O; 37.8%/ A498; 28.3%) for suppressing growth (Fig. 1A). In addition, consistent with the cell viability results, the combination of sunitinib and Rh2 (Caki-1; 23.7%/ 786-O; 14.6%/ A498; 23.0%) resulted in better inhibition of metastasis than in combination with Rg3 (Caki-1; 31.8%/ 786-O; 37.2%/ A498; 36.3%) (Fig. 1C). To evaluate the effectiveness of sunitinib and Rh2 combination therapy, we used the xenograft model to test effects in ccRCC cell lines. The single administrations of sunitinib and Rh2 significantly reduced tumor size and weight, but the effect of the combined administration of sunitinib and Rh2 was more remarkable (Fig. 1D,E). In the immunohistochemistry (IHC) analysis of xenograft tumor sections, the expression of Ki-67 as the proliferation marker was lower in the combination treatment group than in the sunitinib or Rh2 treatment group (Fig. 1F). These results indicate that ginsenosides, especially Rh2, effectively enhance the anti-cancer effects of sunitinib.

### Cell cycle arrest in ccRCC cell lines is increased by combination treatment with sunitinib and ginsenoside

**Figure 2:**
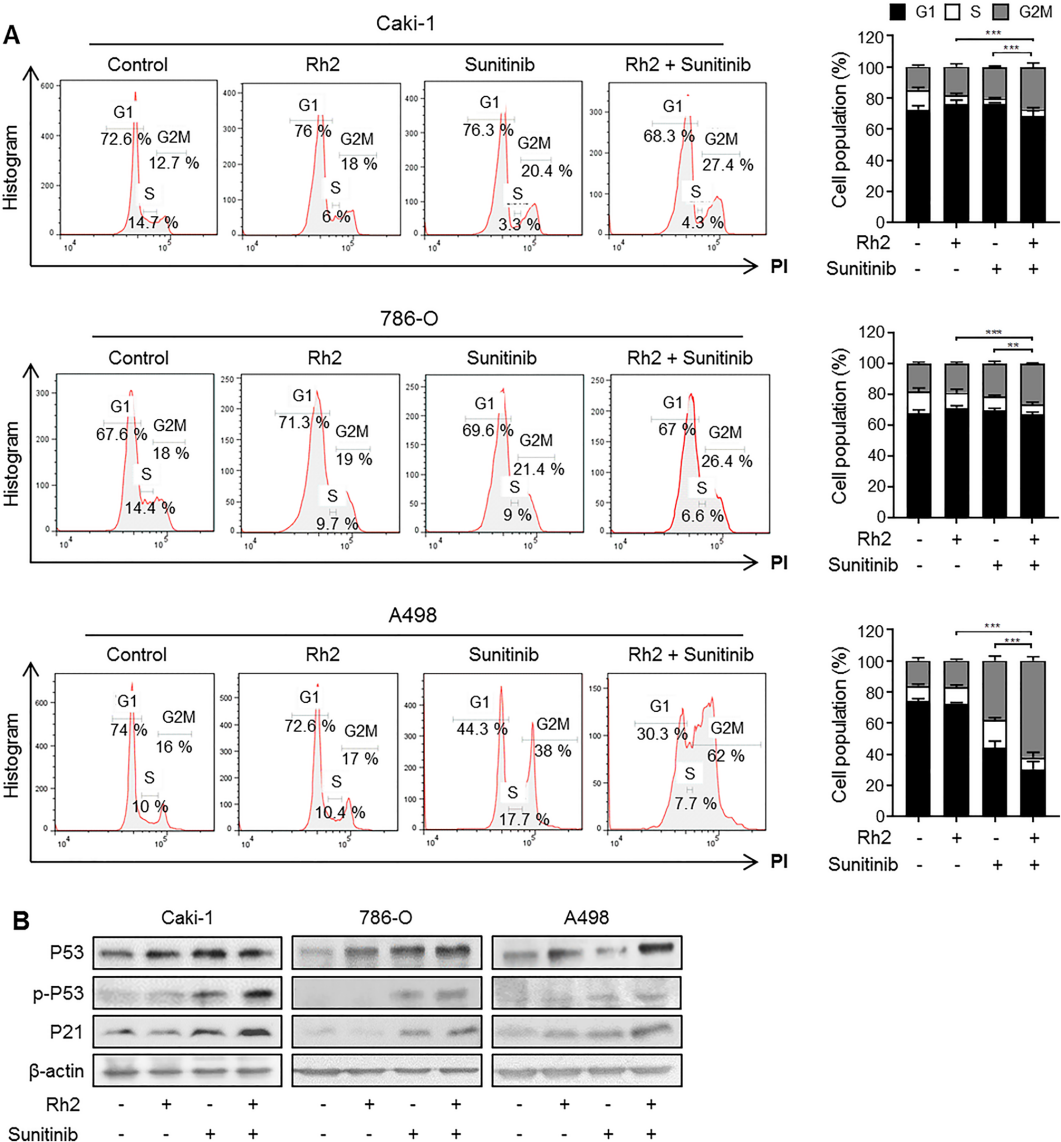
Combination treatment of sunitinib and ginsenoside increases cell cycle arrest in ccRCC cell lines. (**A**) Cells were treated with ginsenoside Rh2 (10 μM) and sunitinib (10 μM) and analyzed using propidium iodide staining detected by flow cytometry. Quantification of the cell cycle was performed. Perform at least three biological repeats (n = 3). (**B**) Protein levels of p-P53 (p-S15), P53, and P21 were detected by western blot. β-actin was used as a loading control. (n = 3).

To evaluate whether the combination of sunitinib and ginsenoside has a synergic effect on inhibition of ccRCC proliferation, we assessed the cell cycle using flow cytometry. Cell cycle arrest triggered by sunitinib/ginsenoside Rg3 or Rh2 was determined using PI staining. In flow cytometry analysis, each ginsenoside induced G1 arrest and sunitinib induced G2M arrest, but co-treatment by Rh2 and sunitinib further enhanced G2M phase arrest (Figs. 2A and S2A). We conducted western blot analysis to evaluate the protein levels of the cell cycle arrest marker. Phosphorylation of P53 on serine 15 residue, which is a primary response to DNA damage, is important for P53 activation^21,22^ and expression of P21^23,24^. The western blot data indicated that the expression levels of p-P53 and P21 increased in the single drug group compared to the control group, and increased in the combination treatment group compared to the single drug or control group (Figs. 2B and S2B–D). These data showed that ginsenoside Rg3 and Rh2 significantly promoted sunitinib-induced cell cycle arrest.

### Combined administration of ginsenoside Rh2 and sunitinib increase DNA damage, activating ATM and ATR pathways

**Figure 3:**
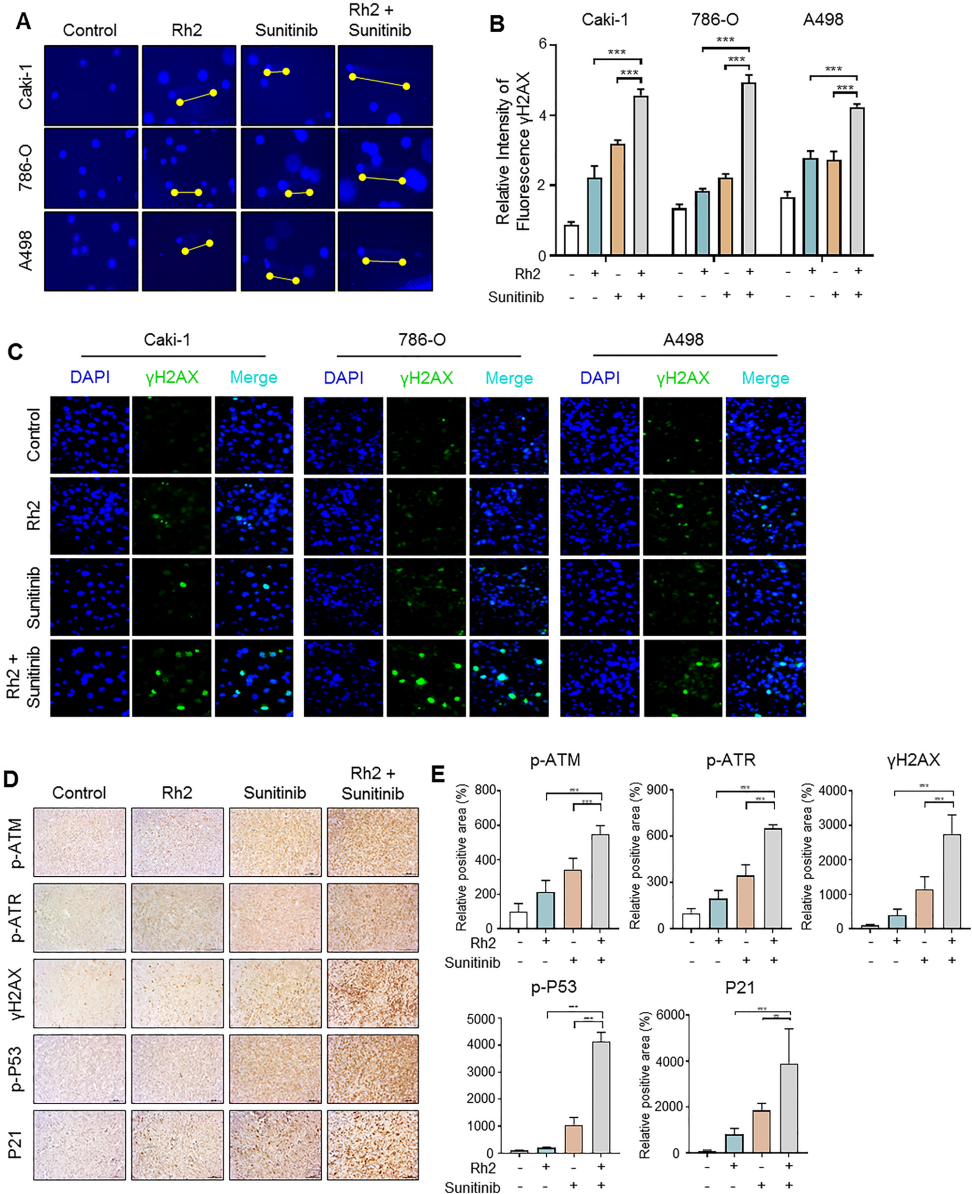
The combination treatment of ginsenoside and sunitinib increases DNA damage response by activating ATM and ATR in ccRCC. (**A**) Comet assay induced by ginsenoside Rh2 and/or sunitinib. The tails indicate DNA damage. Representative images of cell nuclei stained with Hoechst blue after electrophoresis at alkaline pH. (× 400 magnification). (**B**,**C**) Immunocytochemistry (ICC) of γH2AX (green) showed DNA damage after treatment of Rh2 and sunitinib. Counterstaining with DAPI (blue) was conducted to visualize the nuclei. (× 400 magnification). (**D**,**E**) IHC staining for p-ATR, p-ATM, γH2AX, P53, and P21 proteins in A498 xenograft treated with Rh2/ sunitinib. We quantified the protein levels identified in five mice tissues per group (*p < 0.05, **p < 0.01, ***p < 0.001). (× 200 magnification).

In order to detect DNA damage, a major cause of cell cycle arrest, we performed a comet assay to measure the length of the DNA tail, including broken DNA fragments or damaged DNA. In the co-treatment group, a remarkably long DNA tail was observed compared with Rh2 or sunitinib treatment (Fig. 3A). Additionally, we examined the expression of γH2AX, a marker of DNA damage,^25^ by immunocytochemistry (ICC). The fluorescence intensity of γH2AX (green) was significantly higher in the co-treatment group than in the single treatment group (Fig. 3B,C). Next, to determine whether there was a direct link between DNA damage and cell cycle arrest due to drug treatment, we performed IHC using tissue from xenograft tumors. The DNA damage response (DDR) signaling pathway organized by the ATM and ATR kinases is the key regulator of the cellular process networks^26^, and phosphorylation of ATM and ATR by DDR signaling activates P53 and P21 to induce cell cycle arrest^27^. In xenograft tissues, the activities of ATM, ATR, and γH2AX, as well as P53 and P21, were significantly increased in the co-treatment group compared to Rh2 or sunitinib treatment groups (Fig. 3D,E). In the tissue obtained through this xenograft, the increasing pattern of γH2AX by the combination of Rh2 and sunitinib is consistent with the results of the ICC using ccRCC cell lines (Fig. 3B,E). These results indicate that ginsenoside Rh2 promotes sunitinib-induced DNA damage in ccRCC.

### Ginsenoside Rh2 increases oxidative DNA damage by increasing ROS generation by sunitinib

**Figure 4:**
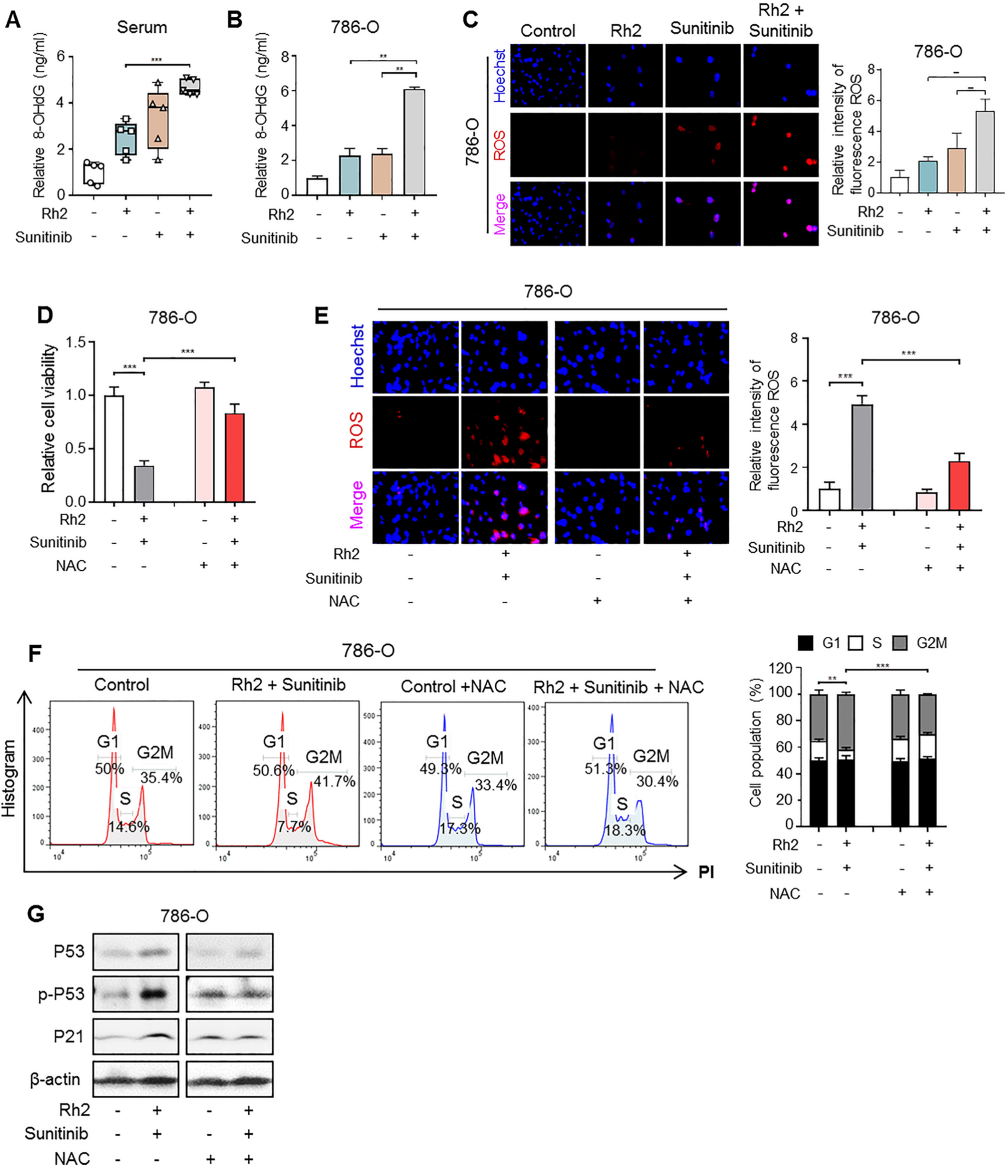
ROS generated by ginsenoside and sunitinib increases levels of 8-OHdG, an oxidative DNA damage marker, in 786-O cells. (**A**) Levels of 8-OHdG in serum samples of xenograft mice were measured by competitive ELISA (***p < 0.001). (n = 5). (**B**) 8-OHdG levels of 786-O cells treated with Rh2 and sunitinib (**p < 0.01). Perform at least three biological repeats (n = 3). (**C**) Fluorescent images of ROS (red) were evaluated in live cells treated with Rh2 and sunitinib. Hoechst (blue) was used as a counter stain. (400 × magnification). (**D**) Changes in cell viability when NAC, a ROS inhibitor, is administered with Rh2 and sunitinib. NAC (5 mM), Rh2 (10 μM), and sunitinib (10 μM) (***p < 0.001). (n = 3). (**E**) ROS induced by sunitinib and Rh2 was alleviated by treatment with NAC. Measurements were taken 24 h after administration of the drug with a fluorescence microscope. (400 × magnification). (**F**) Cell cycle analysis by flow cytometry of 786-O cells treated with Rh2, sunitinib, and NAC. Histogram data from the cell analysis are quantified and graphed. (n = 3). (**G**) Western blot analyses to evaluate the expressions of the indicated proteins in 786-O cells that were treated with Rh2, sunitinib, and NAC. We confirmed that the protein expressions of cell cycle arrest markers were reduced by NAC. (n = 3).

Oxidative damage is the main cause of damage to DNA^28^. To examine whether the combination of Rh2 and sunitinib directly induces oxidative DNA damage, we measured the levels of 8-hydroxy-2'-deoxyguanosine (8-OHdG), a marker of oxidative DNA damage, in xenograft serum and ccRCC cell lines. Compared to the Rh2 treatment group, the sunitinib and Rh2 combination treatment groups had higher levels of 8-OHdG (Fig. 4A). The same results were shown in ccRCC cell lines (Figs. 4B and S3A). For serum, the sunitinib and combination groups showed no significant results but levels were remarkably increased in the cell lines. Oxidative stress that causes DNA damage reflects increases in levels of ROS^29^. ROS levels were measured with a fluorescence microscope, revealing significant increase of ROS level in the combination group (Figs. 4C and S3B). We investigated the transcriptional level of three common genes (NFE2L2, NFKB1, and HIF1A gene) increasing by ROS generation. In our qRT-PCR data, the mRNA level of NFKB1 and HIF1A increased in the combination group, but not the mRNA level of NFE2L2 (Fig. S4C). In addition, N-acetyl-L-Cysteine (NAC), a ROS inhibitor, was used to determine whether oxidative stress by Rh2 and sunitinib can be alleviated. We confirmed that cell viability decreased after combination treatment with Rh2 and sunitinib, but recovered through treatment with NAC (Figs. 4D and S3C). The ROS level in the combination group decreased due to NAC, and as a result cell cycle arrest was also alleviated (Figs. 4E,F, S3D and S4A). In addition, the protein levels of cell cycle arrest markers decreased after NAC treatment (Figs. 4G and S4B). Taken together, our results indicate that a combination of Rh2 and sunitinib, which has been confirmed to induce cell cycle arrest by increasing ROS, is a feasible novel ccRCC treatment method.

## Discussion

**Figure 5:**
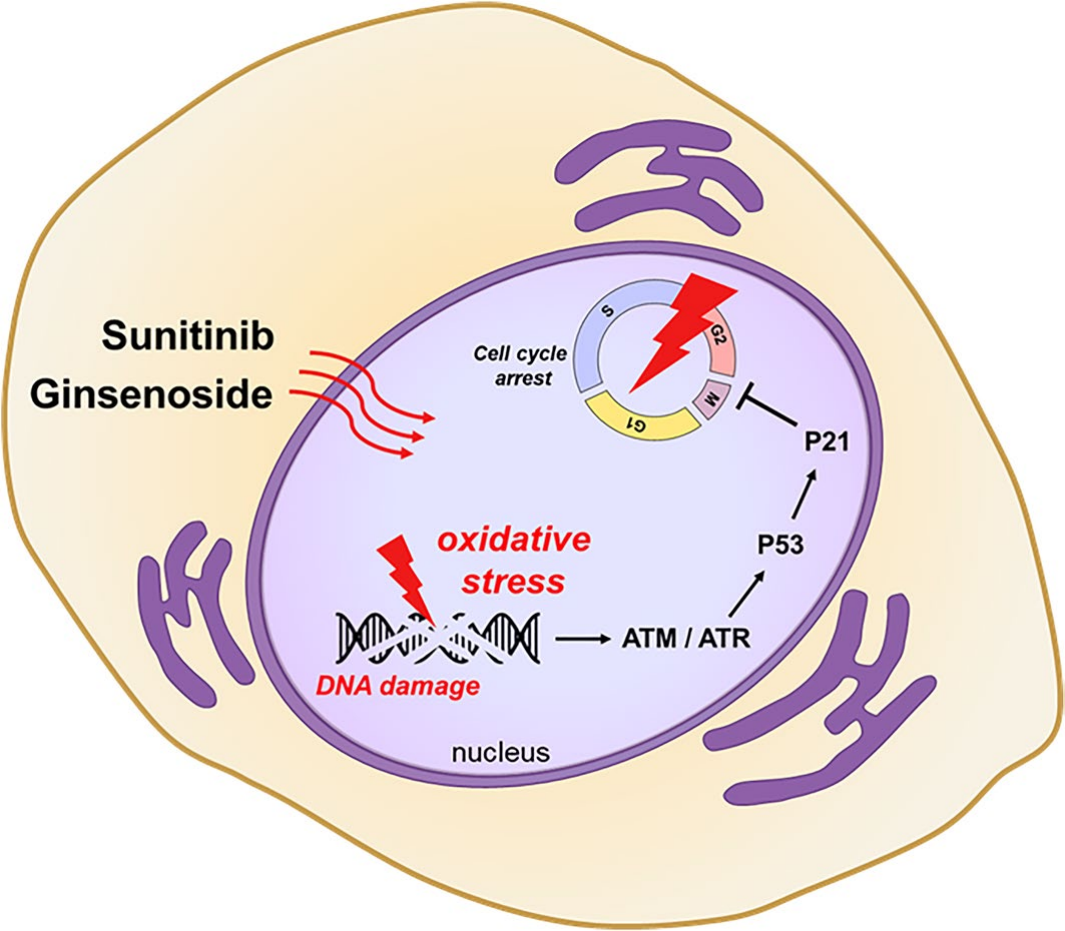
Ginsenoside increases DNA damage due to sunitinib to induce cell death by cell cycle arrest. Combination treatment of sunitinib and ginsenoside Rh2 induces oxidative stress in ccRCC. The DNA damage by oxidative stress arrests the cell cycle and inhibits cell growth of ccRCC.

In the present study we demonstrated that ginsenoside enhances the sensitivity of sunitinib to inhibit the proliferation of renal cell carcinoma cells via induction of cell cycle arrest. Ginsenoside Rg3 and Rh2 induced G1 phase arrest, while co-treatment with sunitinib further increased G2/M arrest in ccRCC cells. These cell cycle analyses confirmed that the co-treatment of ginsenoside and sunitinib enhances cell cycle arrest. We measured DNA damage among the causes of cell cycle arrest and found that DNA damage by Rh2 and sunitinib increased by detecting changes in comet assay and γH2AX expression. Moreover, we found that the protein expressions of p-ATM and p-ATR, which are activated first when DNA damage occurs, were expressed to a greater degree in co-treatment tissues than in single-drug treatment tissues. Increases of 8-OHdG, an oxidative DNA damage marker, was found in both the serum and cell lines of the group that was treated with both drugs. The ROS-induced oxidative stress increased significantly in the combination group. To determine whether Rh2 and sunitinib reliably induce ROS, NAC was used as a ROS inhibitor. We confirmed that cell viability and cell cycle in samples treated with both drugs recovered after NAC treatment. Therefore, ginsenoside increases the oxidative DNA damage induced by sunitinib, which causes cell cycle arrest to inhibit cell growth (Fig. 5).

DNA damage can be induced by various external stimuli, including oxidative stress, UV exposure, and chemotherapeutic drugs^30^. In addition, metabolic reactions caused by various stresses result in three types of DNA damage: DNA adducts, oxidative DNA damage, and dNTP pool alterations^31^. ROS produce oxidative DNA damage. Interestingly, ginsenoside is known to exhibit the function of antioxidants in inflammation disease^32^, while it causes oxidative damage to inhibit the cell growth in cancer^33–36^. The reaction of ROS with DNA mainly comes about due to reactions of OH with pyrimidines, purines, or sugars in DNA, and one of the most frequent oxidative DNA lesions is 8-hydroxy-2-deoxyguanosine (8-OHdG)^37^. Therefore, 8-OHdG is known to be a biomarker for oxidative damage of DNA^38,39^. Also, a recent study reported the correlation between sunitinib and oxidative stress in RCC^40^. Although the study differs from our research in that papillary RCC, not ccRCC, is the model and sunitinib arrests the G1 phase not the G2M phase, it is consistent with our results that sunitinib inhibits cancer growth by inducing oxidative stress in RCC. We confirmed the level of 8-OHdG in the serum obtained mice xenograft model and in ccRCC cell lines and observed that the co-treatment group exhibited greater increases in 8-OHdG than the single treatment groups. Interestingly, H2AX is activated by DNA damage phosphorylates ATM and ATR, while inversely activating ATM and ATR phosphorylate H2AX^41^. Since both ATM (involved in double-strand breakage) and ATR (involved in single-strand breakage)^42^ are increased by both drugs, ginsenoside and sunitinib comprise two ways to cause DNA breakage.

To ensure that damaged DNA is not propagated to the next generation, cell cycle regulation stabilizes conditions necessary for cell growth and homeostasis^43^. Three checkpoints, G1, S, and G2M, phase control DNA replication and cell death in cancer, and are controlled by P53, a key regulator alongside CDK and P21^44,45^. In Fig. 2, cell cycle arrest was triggered to prevent DNA damage induced by ginsenoside and sunitinib. Down-regulation of CDK and up-regulation of P21 inhibit progression through cell cycle checkpoints, resulting in cell cycle arrest^46^. These changes in the expressions of cell cycle arrest markers (Fig. 2B) suggest that Rh2 and sunitinib induced cell cycle arrest by oxidative DNA damage in ccRCC.

Co-treatment with ginsenoside and sunitinib in ccRCC caused DNA damage and cell cycle arrest, and thereby suppressed the growth of cancer. We propose that ccRCC can be effectively treated by increasing the sensitivity of sunitinib, an existing treatment for ccRCC, through co-treatment with ginsenoside, a natural product. Furthermore, more extensive studies such as orthotopic xenografts or patient-derived xenografts are needed to increase the potential of combination therapy using sunitinib and ginsenoside Rh2 for ccRCC patients by better understanding the profound effects on local tumor growth and predictive response values of ccRCC.

## Methods

### Cell culture

Caki-1 and 786-O cells (ATCC, Manassas, VA, USA) were cultivated in RPMI medium including L-glutamine (Sigma-Aldrich, #R8758, St. Louis, MS, USA) and A498 (ATCC) was cultivated in DMEM medium containing 4.5 g/L glucose (Sigma-Aldrich, #D6429). Media were added to 10% fetal bovine serum (FBS) (Sigma-Aldrich, #TMS-013-BKR) and 1% antibiotic–antimycotic (GIBCO, #15240062, Waltham, MA, USA). Cells were maintained at 37 °C under 5% CO_2._

### Drugs

Ginsenoside Rg3 (Sigma-Aldrich #SML0184), Rh2 (Sigma-Aldrich #73658), and sunitinib malate (Sigma-Aldrich #PZ0012) were melted in DMSO (Sigma-Aldrich) at a concentration of 10 mM. *N*-acetyl-l-Cysteine (NAC) was used as a ROS inhibitor (Sigma-Aldrich, #A9165-5G) by melting in DMSO to a concentration of 5 M and heating.

### Cell proliferation assay

The viability of Caki-1, 786-O, and A498 cells was assessed using the EZ-CYTOX (DoGenBio, #EZ-1000, Seoul, Korea). All three cell lines were seeded in 96-well plates (1 × 10^4^ cells/well). After incubation overnight, the cells were treated with different concentrations of ginsenoside Rg3, Rh2, and sunitinib for 24 h. Then EZ-CYTOX solution was supplemented to each well. Absorbance at a wavelength of 450 nm was detected by a Microplate reader. The cell viability rates were calculated, and graphs were generated.

### Matrigel invasion assay

Caki-1, 786-O, and A498 cells were plated in a serum-free medium for 24 h. A total of 3 × 10^5^ cells containing fresh media were seeded into an 8 μM transparent PET membrane (FALCON, #353097, Corning, NY, USA) and placed in 24 well plates containing 20% FBS fresh media. Cells were allowed to invade for 24 h. Samples were fixed with 3.7% formaldehyde and then subjected to a permeabilization process with 100% methanol. Samples were dyed with 0.4% crystal violet. Images of the invading cells were captured using a inverted microscope (Nikon, #TS100, Tokyo, Japan) at × 40 magnification.

### Cell cycle assay

First, 5 × 10^5^ cells were fixed in 80% ethanol for 1 h at − 20 °C. Then, the cells were washed twice with phosphate-buffed saline (PBS), and 500 μl PI/RNase staining buffer (BD Pharmingen, #550825, San Diego, California, USA) was added. The samples were incubated at RT for 20 min in a dark environment and analyzed by flow cytometry.

### Immunohistochemistry (IHC)

Paraffin-embedded tumor tissue specimens were sliced into 3-μm-thick sections and mounted onto slides. Then, the slides were subjected to de-paraffinization (xylene) and rehydration (ethanol). Slides were submerged in boiling citrate buffer pH 6.0 for antigen retrieval and incubated with specific primary antibodies overnight at 4 °C. Subsequently, the sections were incubated with secondary antibodies (1:200) after washing with PBS. After staining with diaminobenzidine (DAB), the tumor sections were visualized under a optical microscope (Leica DM s00B microscope, Leica Microsystems Inc., Buffalo Grove, IL, USA) at × 200 magnification. In this study, the tumor sections were stained with phospho-ATM (Ser 1981, 1:100) (Abcam, #ab81292, Cambridge, UK), phospho-ATR (Ser 428, 1:100) (Abcam, #ab178407), P53 (1:100) (Abcam #ab1101), phospho-P53 (Ser 15, 1:100) (Cell signaling technology, #9284S, MS, USA), P21 (1:200) (Abcam, #ab109520), and ki-67 (1:250) (Abcam, #ab91742) to assess the expressions of proteins in ccRCC cell lines. Quantification was scored by the product of intensity and percentage of staining.

### Xenograft model and treatments

Female BALB/c nude mice (4 weeks old) were purchased from Orient Bio (South Korea) and were housed under specific pathogen-free conditions. All animal experimental protocols were approved by the Hanyang University Institutional Animal Care and Use Committee (2020-0104A). All procedures related to the in vivo experiments and animal care were carried out in accordance with the approved guidelines. The study is compliant with the ARRIVE guideline 2.0.

To establish the xenograft model, 1 × 10^7^ A498 cells were injected subcutaneously into the side regions of nude mice. The mice were randomized into four groups (n = 5) and administered drugs when the tumor volume reached approximately 300 mm^3^. Sunitinib (10 mg/kg) was administered orally daily, and ginsenoside Rh2 (10 mg/kg) was applied by intraperitoneal injection three times a week. For the combination treatment, the two drugs were administered together. Body weights and tumor volumes were measured three times per week using calipers. After 4 weeks of treatment, the mice were sacrificed and tumor tissues were harvested and fixed in formalin for IHC staining.

### Western blot analysis

Caki-1, 786-O, and A498 cells were treated with sunitinib (10 μM) and ginsenoside Rh2 (10 μM) for 24 h. In brief, cells were lysed in lysis buffer and protein was extracted by centrifuge. For immunoblotting, 30 μg proteins was separated by 10–12% SDS-PAGE and transferred from the polyacrylamide gel to the PVDF membrane. The membranes were blocked with 5% skim milk in TBS-T buffer, further incubated with specific primary antibodies phospho-P53 (Ser 15, 1:1000) (Cell Signaling Technology, #9284S), P53 (1:1000) (Abcam #ab1101), and P21 (1:1000) (Abcam, #ab109520) at 4 °C overnight and followed by incubation with secondary antibodies at RT for 1 h. Protein bands were detected by a film using developer and fixer.

### Immunocytochemistry (ICC)

Cells were fixed with 4% paraformaldehyde (Biosesang, #PC2031-100, Gyeonggido, Korea) and permeabilized using 0.1% Triton X-100 in PBS. After sufficient washing with PBS, cells were blocked for 30 min under RT using 10% normal goat serum. Cells were incubated with anti-γH2AX (phospho serine 139, 1:200) antibody (Abcam, #ab81299), followed by secondary antibodies labeled with AlexaFluor488 (anti-rabbit IgG, 1:200) (Invitrogen, #A32731, Waltham, MA, USA). DNA was stained with Hoechst 33342 (1:200) (Thermo scientific, #62249, Waltham, MA, USA). Images were photographed utilizing a fluorescence microscope at × 400 magnification.

### Comet assay

Alkaline comet assay was conducted using a comet assay kit (Biotechne, #4250-050-K, Minneapolis, MN, USA) following the manufacturer’s instructions. DNA was stained with Hoechst 33342 (Thermo Scientific, #62249) and fluorescence images were captured using a fluorescence microscope at × 400 magnification.

### Intracellular ROS production

ccRCC cell lines were incubated in DMEM and RPMI in 24-well plates containing 10% FBS. ROS level was measured by fluorometric intracellular ROS kit (Sigma-Aldrich, #MAK145). After treating with the mix solution, samples were incubated for about 1 h and then treated with Rh2 (10 μM) and sunitinib (10 μM) for 24 h and measured with a fluorescence microscope at × 400 magnification.

### Statistical analysis

Statistical analysis was performed using GraphPad Prism 8. All invitro experiments were repeated at least three biological repeats. Statistical significance was determined by t-test, one-way analysis of variance (ANOVA) and two-way ANOVA. A p-value of less than 0.05 was considered significant.

## Supplementary Information

Supplementary Figure 1.

Supplementary Figure 2.

Supplementary Figure 3.

Supplementary Figure 4.

Supplementary Information.

## Data Availability

The data that supports the findings of this study are available within the article.
