# Supplementary figures and images for: Ginsenoside Rh2 sensitizes the anti-cancer effects of sunitinib by inducing cell cycle arrest in renal cell carcinoma
Source: Sci Rep. 2022 Nov 17;12:19752. doi: 10.1038/s41598-022-20075-0 (PMC9672391; doi:10.1038/s41598-022-20075-0)

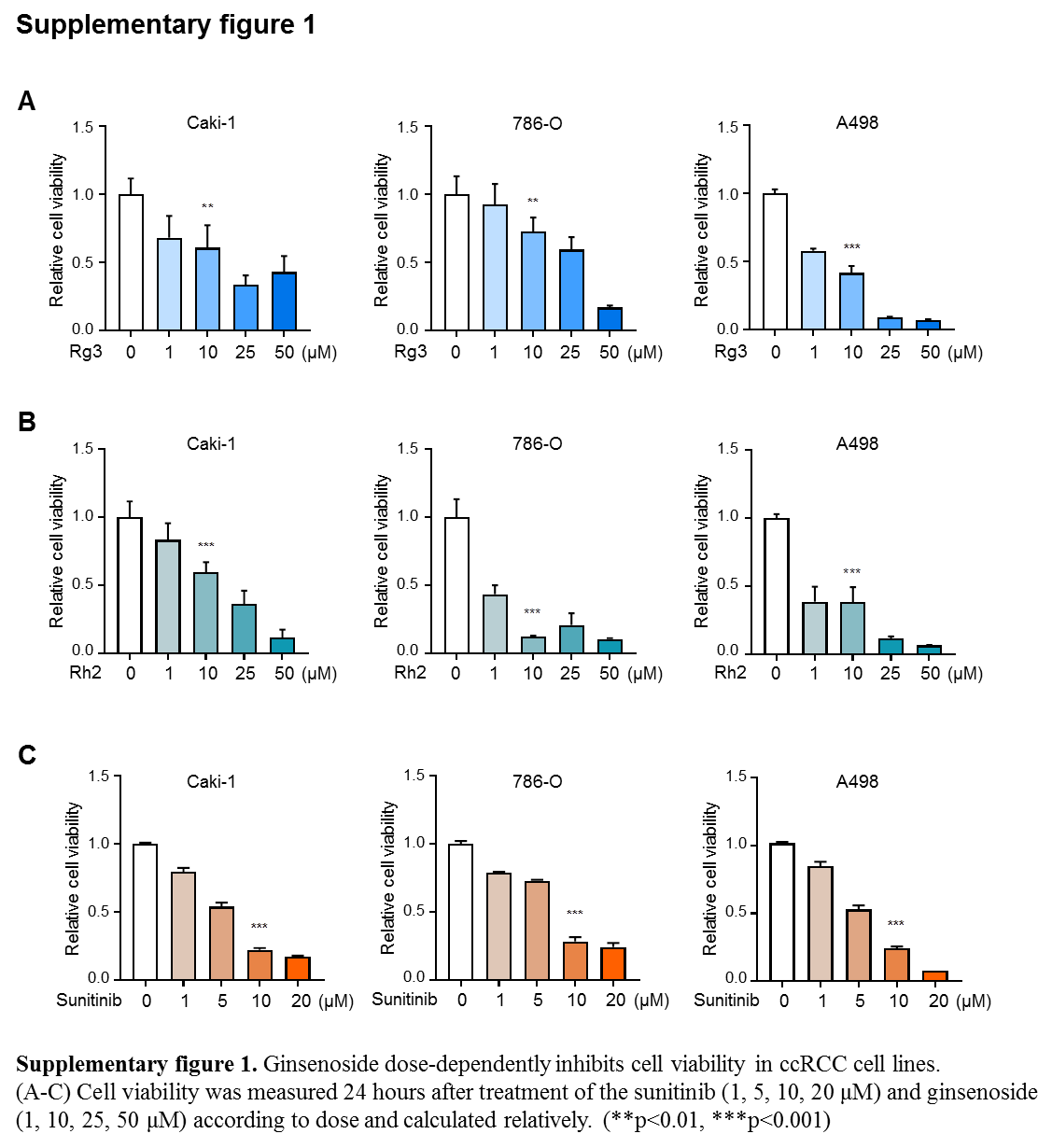

Supplement: Supplementary file 1 — Supplementary Figure 1. [file 41598_2022_20075_MOESM1_ESM.tif]

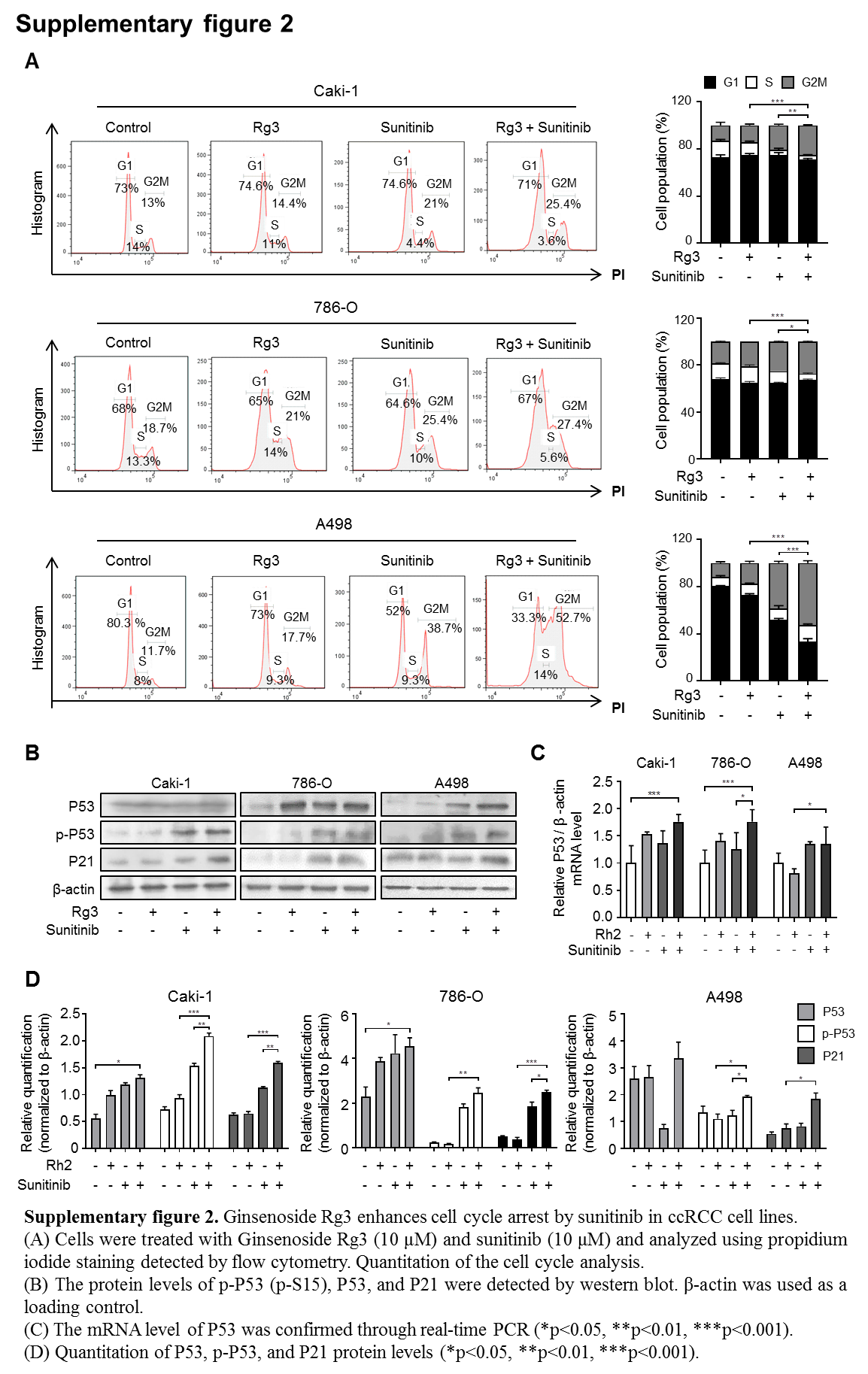

Supplement: Supplementary file 2 — Supplementary Figure 2. [file 41598_2022_20075_MOESM2_ESM.tif]

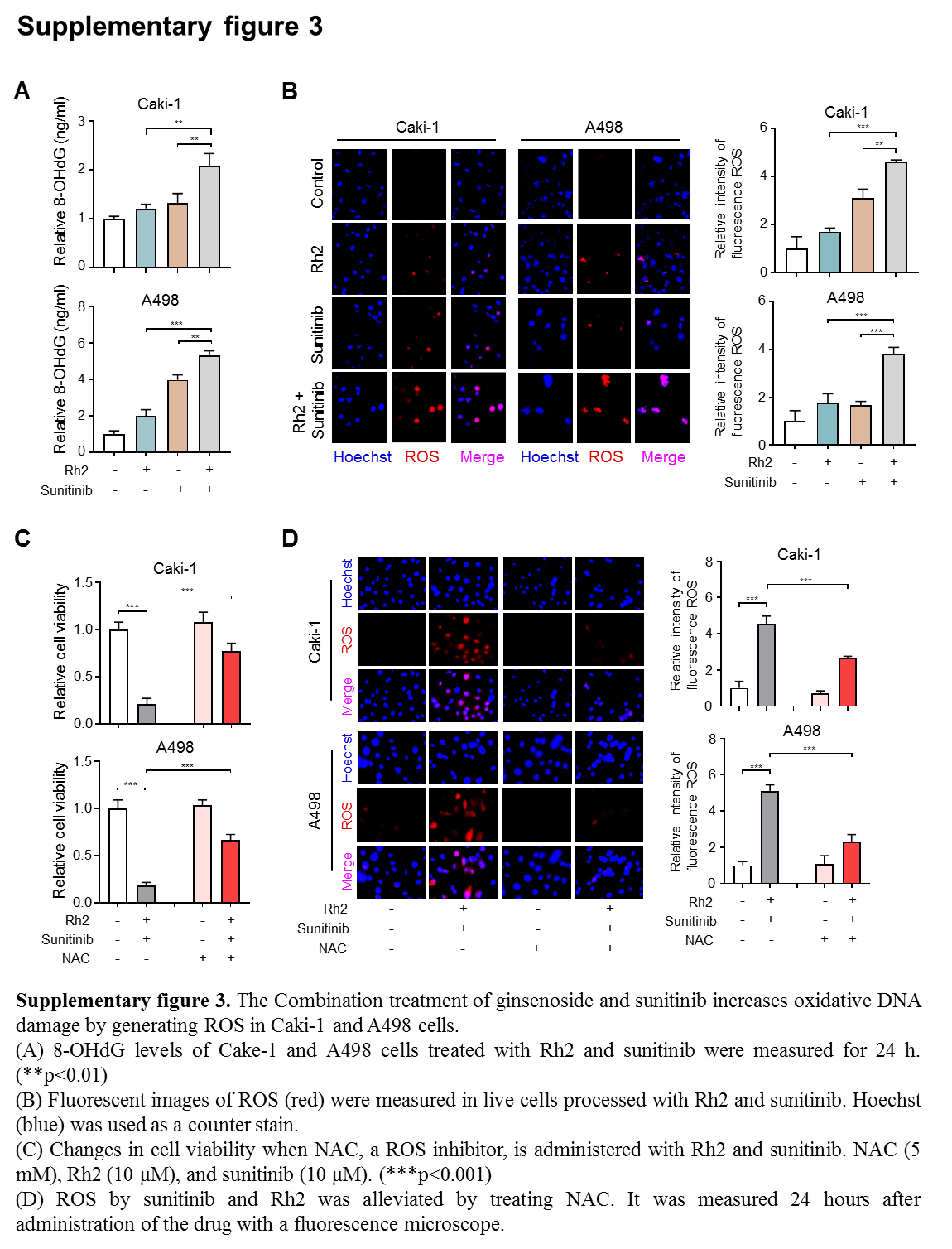

Supplement: Supplementary file 3 — Supplementary Figure 3. [file 41598_2022_20075_MOESM3_ESM.tif]

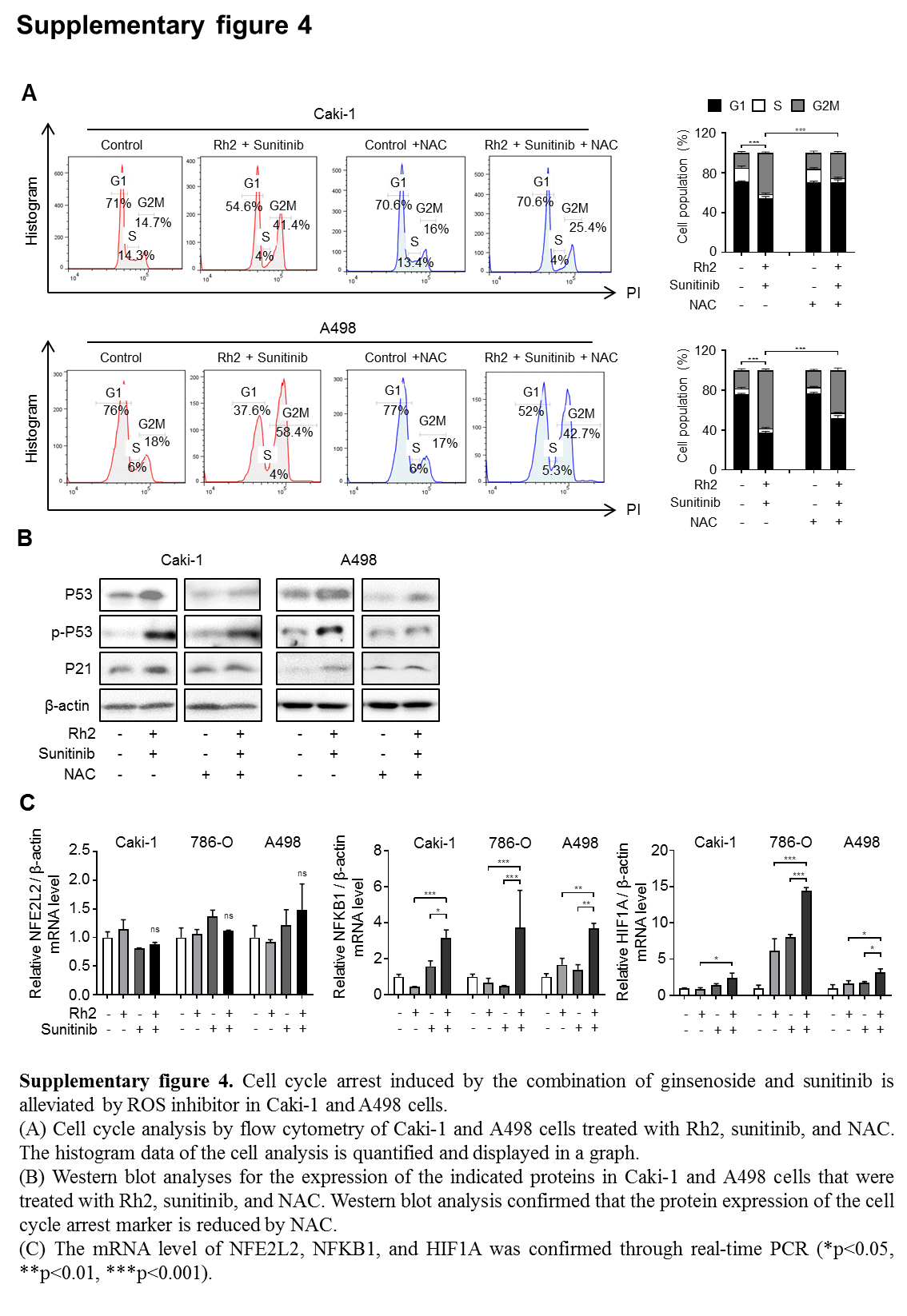

Supplement: Supplementary file 4 — Supplementary Figure 4. [file 41598_2022_20075_MOESM4_ESM.tif]
