# Supplementary material for: Ginsenoside Rh2 sensitizes the anti-cancer effects of sunitinib by inducing cell cycle arrest in renal cell carcinoma
Source: Sci Rep. 2022 Nov 17;12:19752. doi: 10.1038/s41598-022-20075-0 (PMC9672391; doi:10.1038/s41598-022-20075-0)

Figure 2.

P53 (53 kDa)

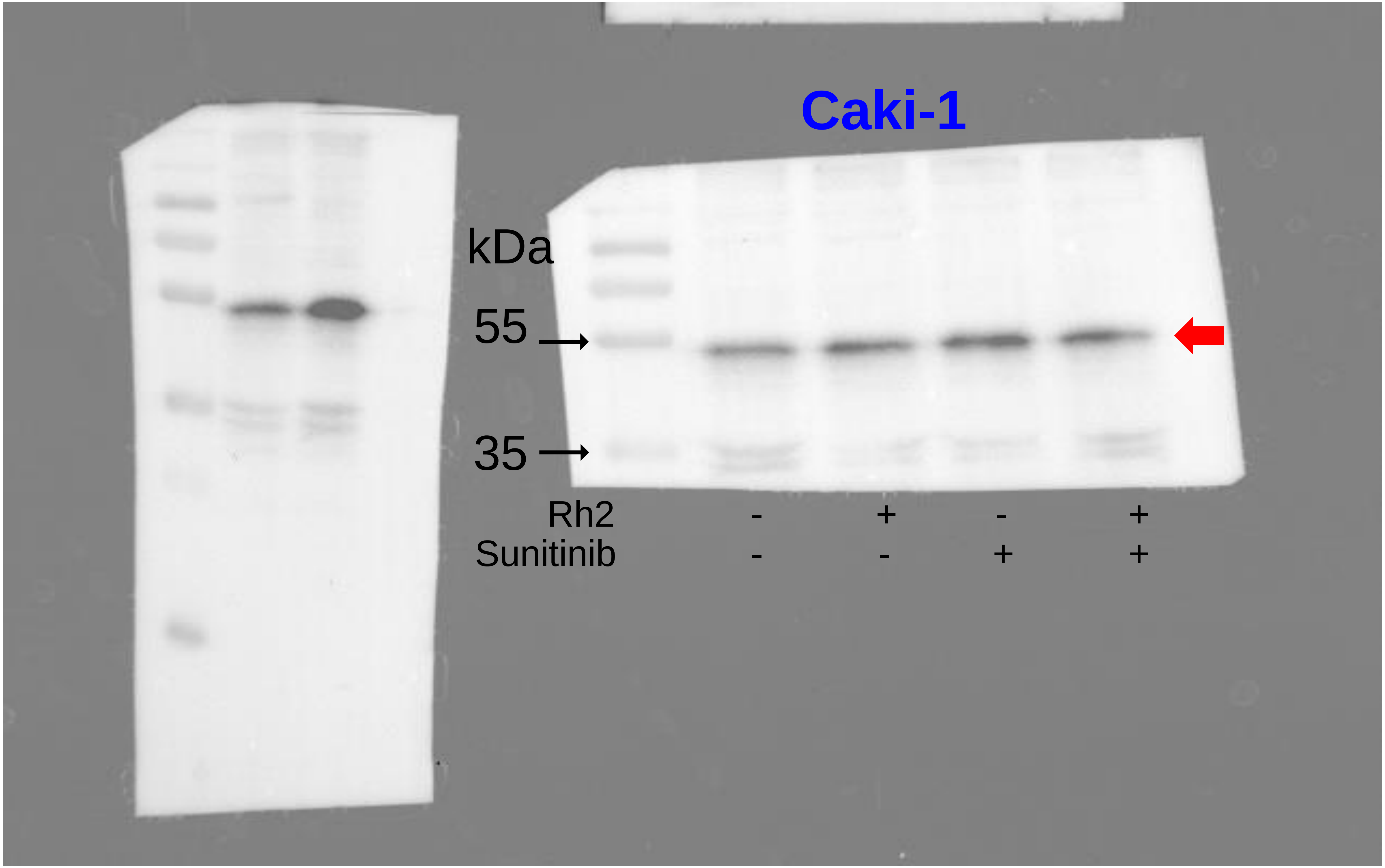

Figure 2.

P53 (53 kDa)

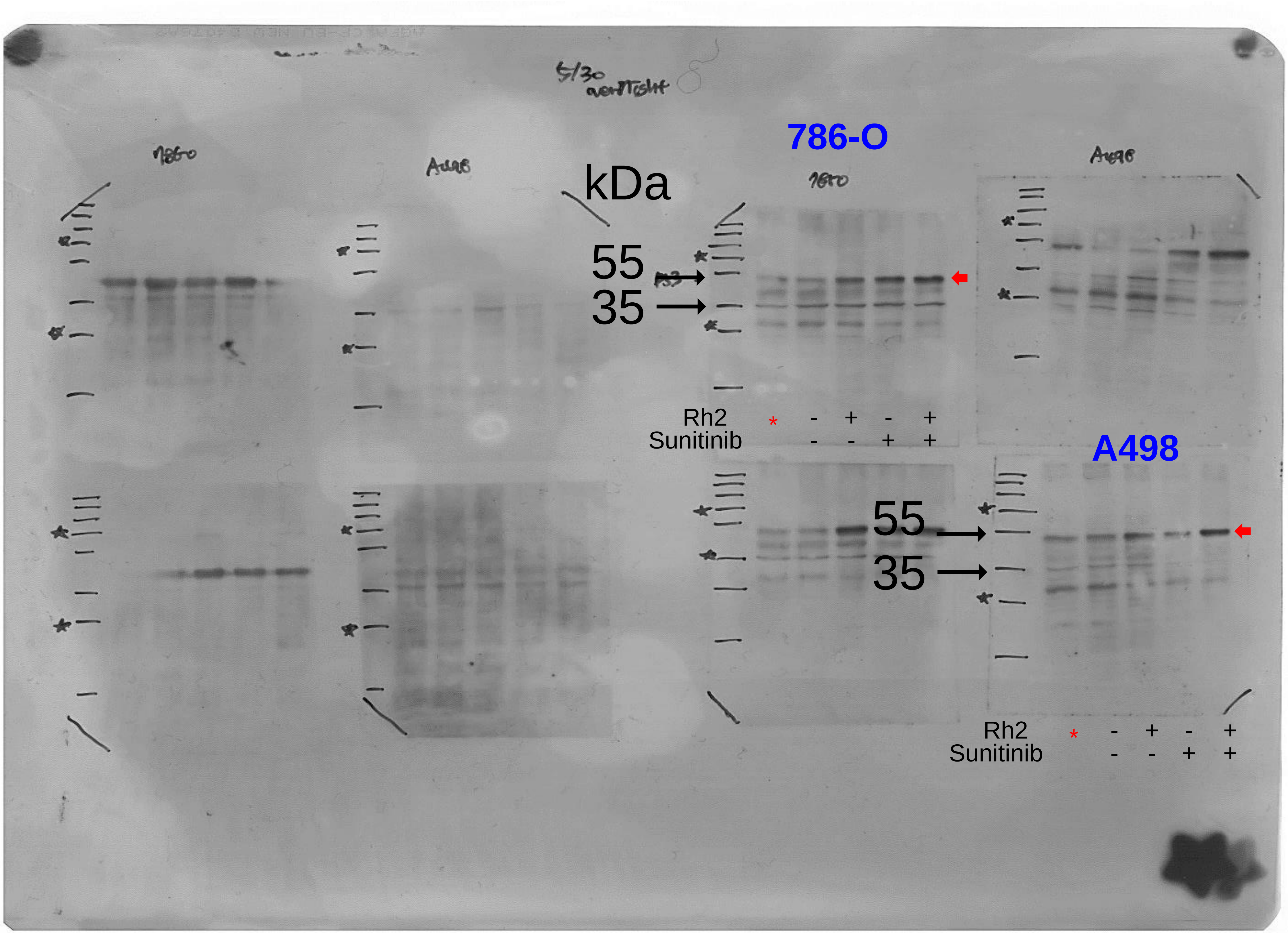

Figure 2.

p-P53 (53 kDa)

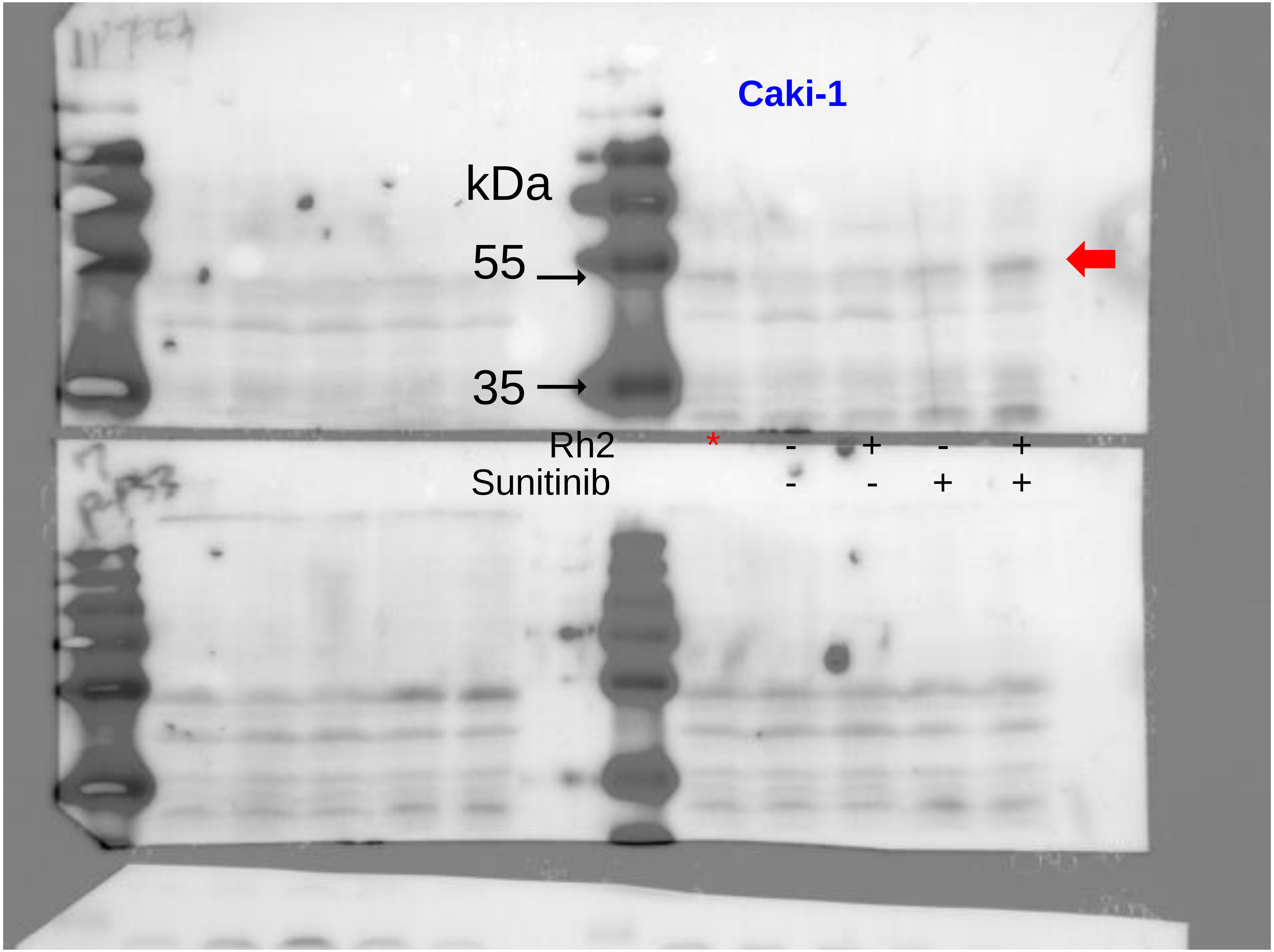

Figure 2.

p-P53 (53 kDa)

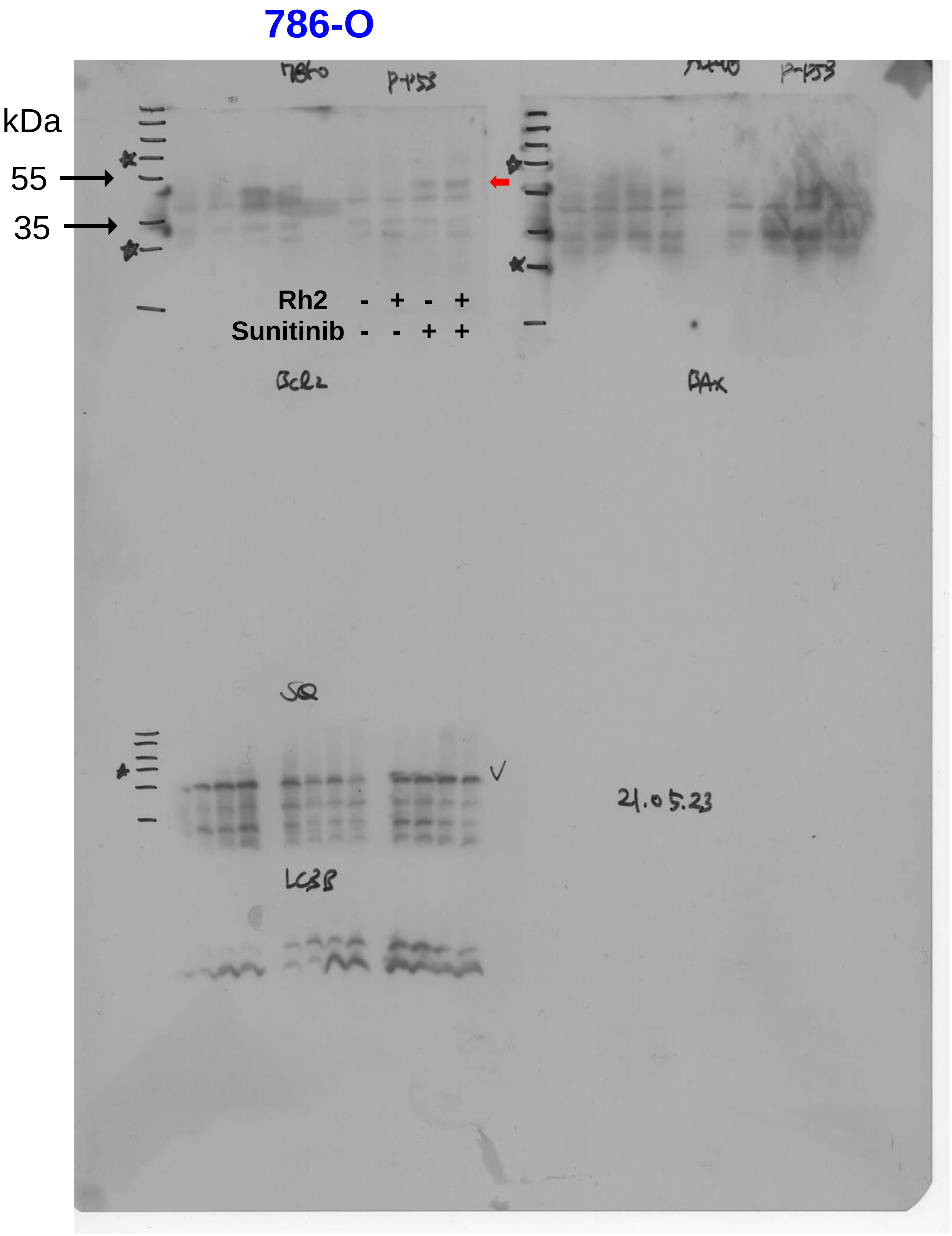

Figure 2.

p-P53 (53 kDa)

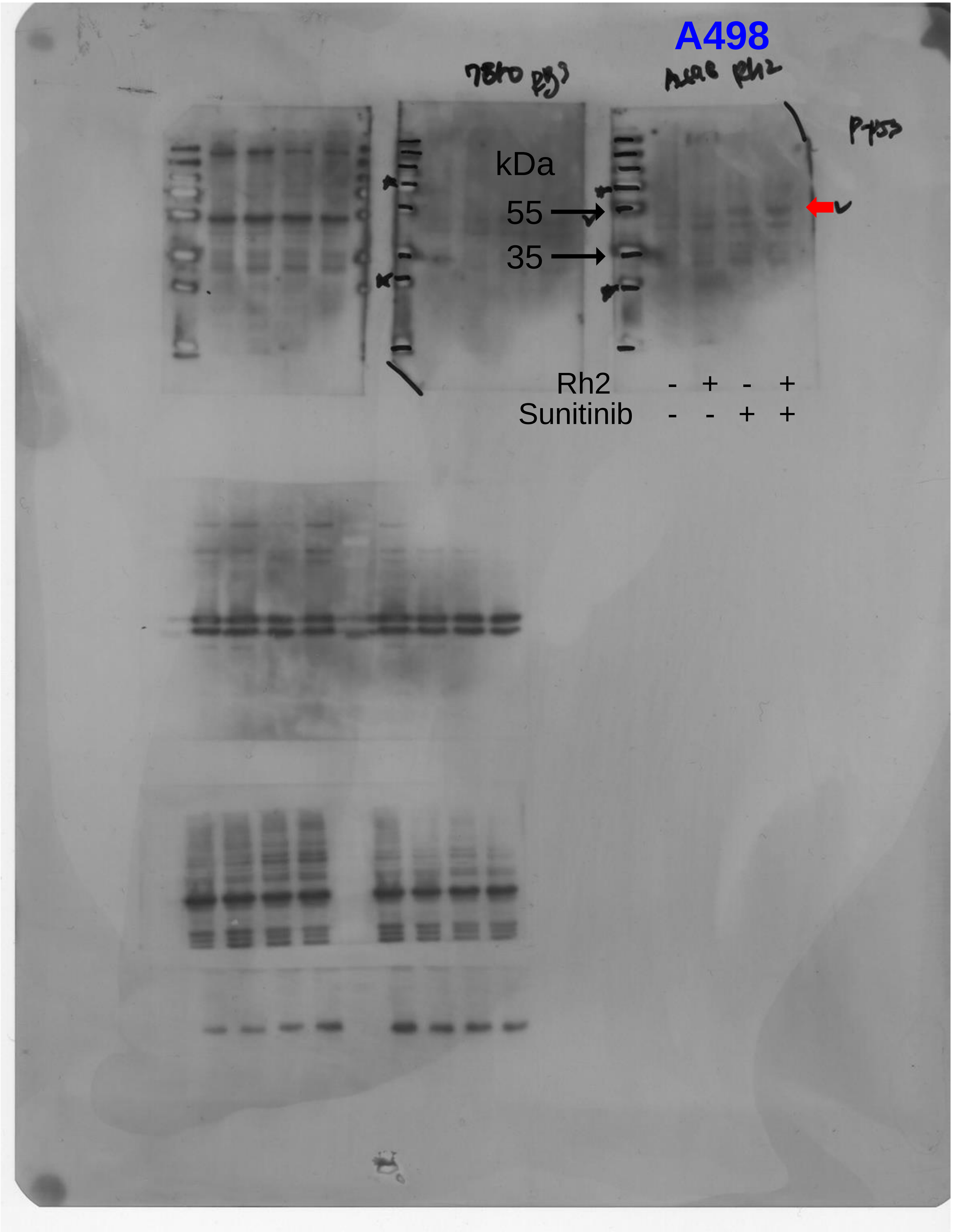

Figure 2.

P21 (21 kDa)

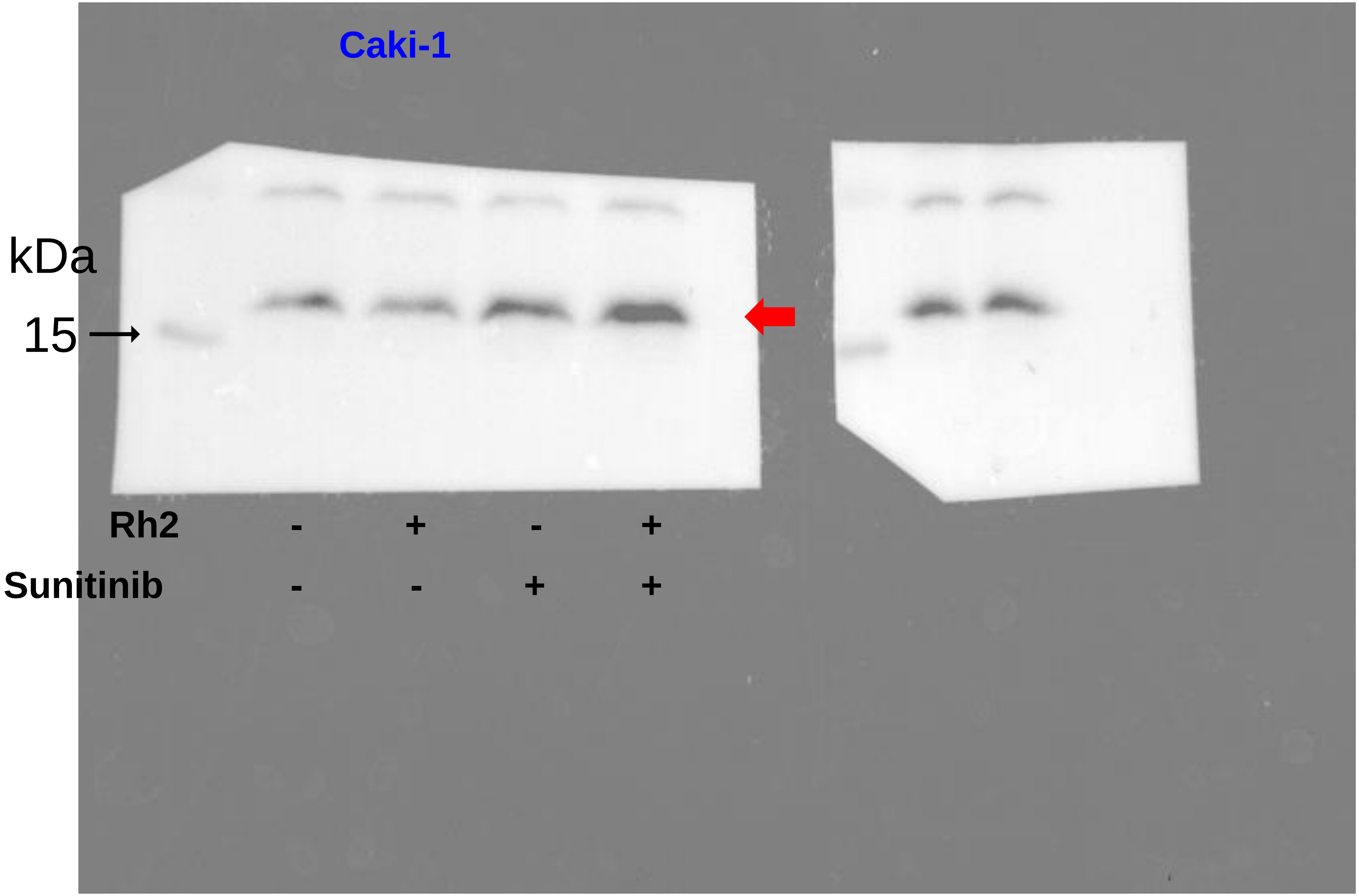

Figure 2.

P21 (21 kDa)

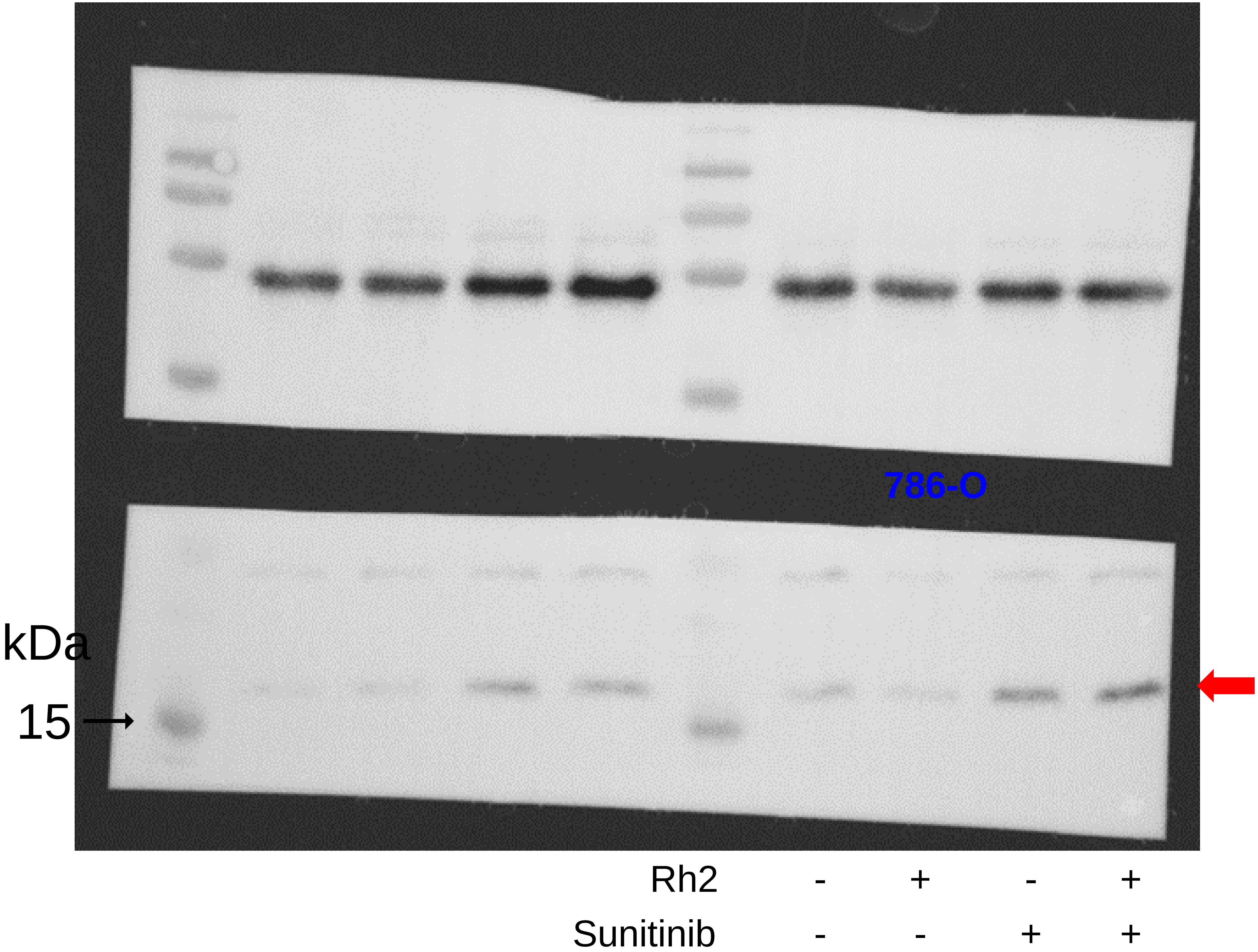

Figure 2.

P21 (21 kDa)

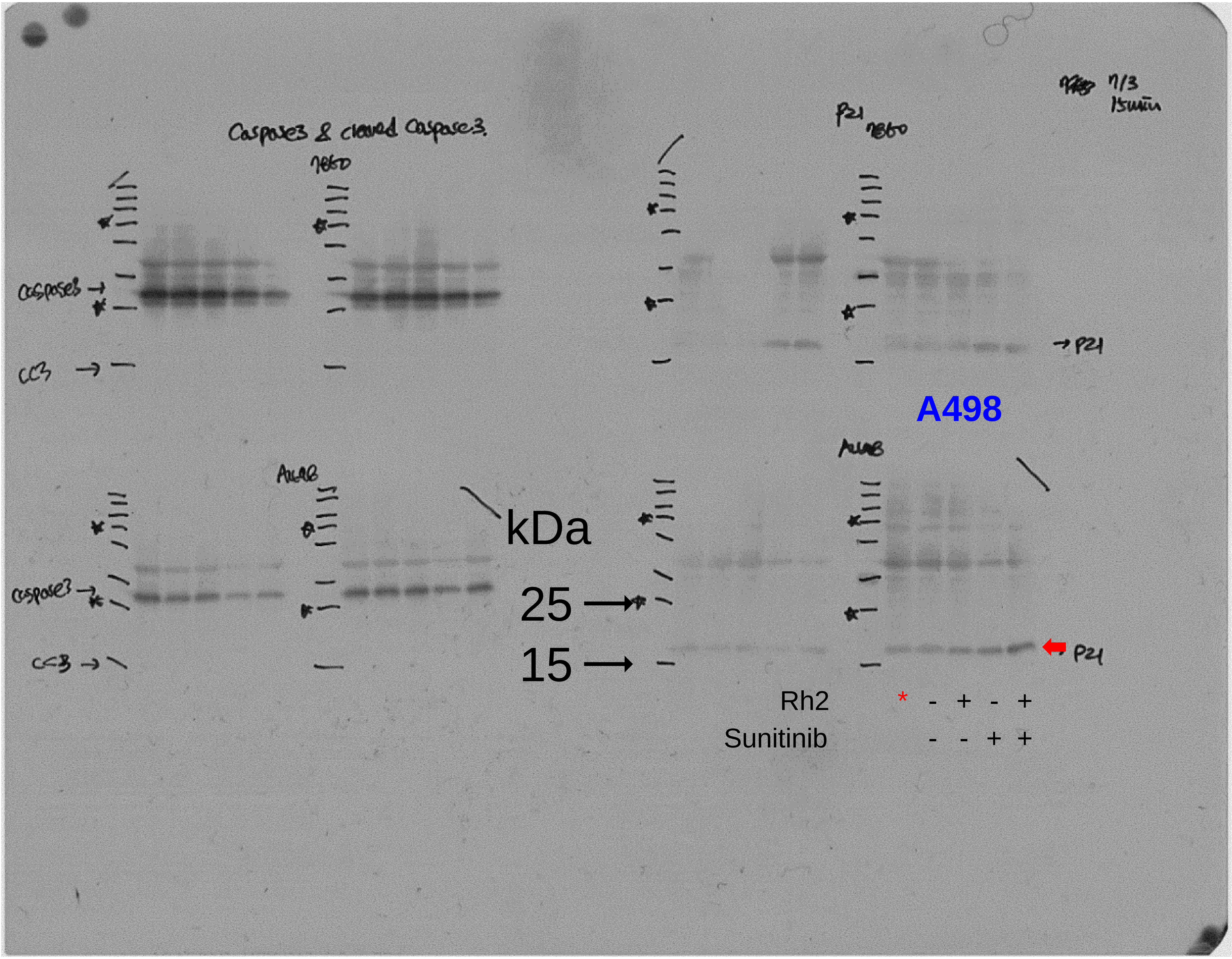

Figure 2.

β actin (43 kDa)

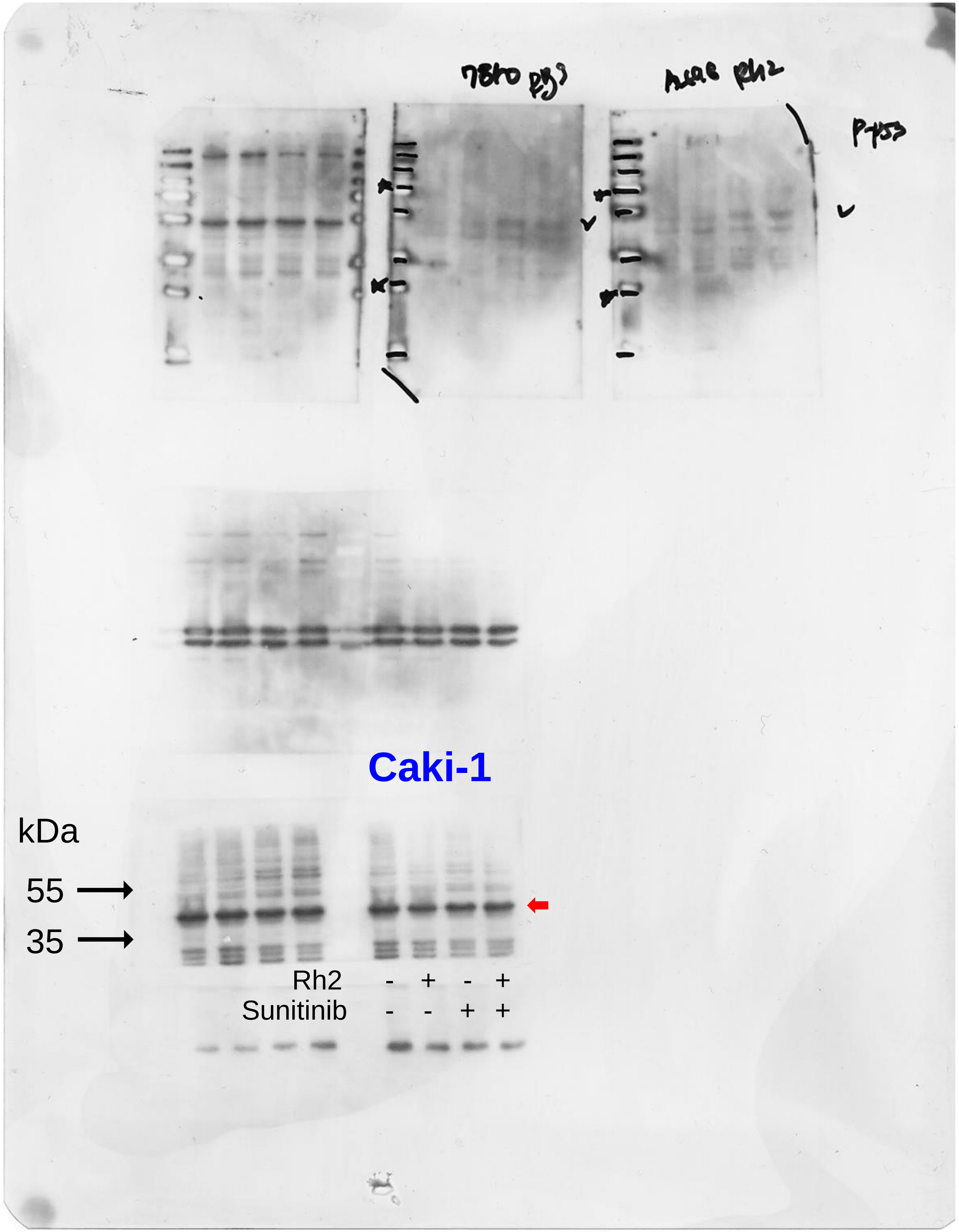

Figure 2.

β actin (43 kDa)

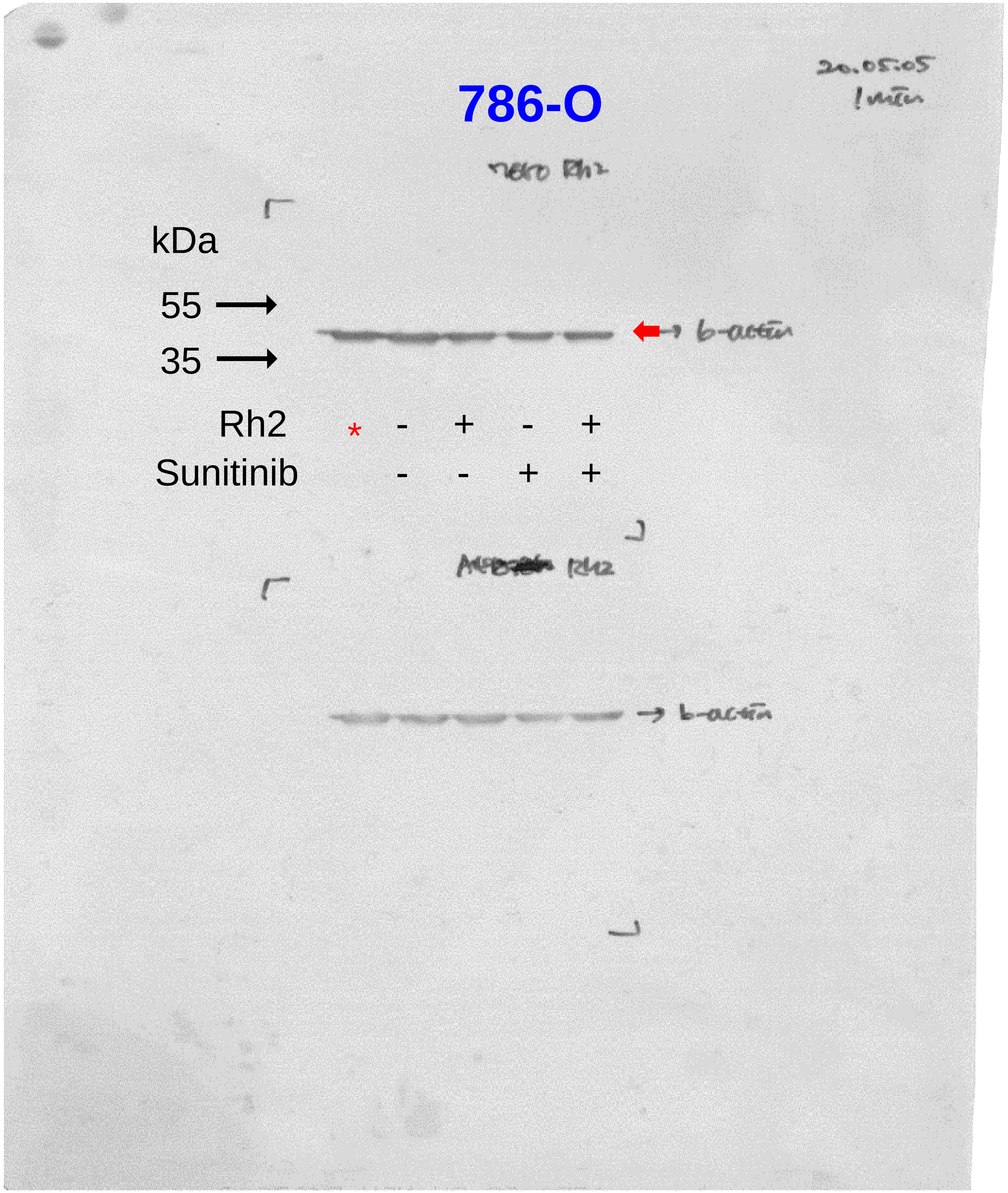

Figure 2.

β actin (43 kDa)

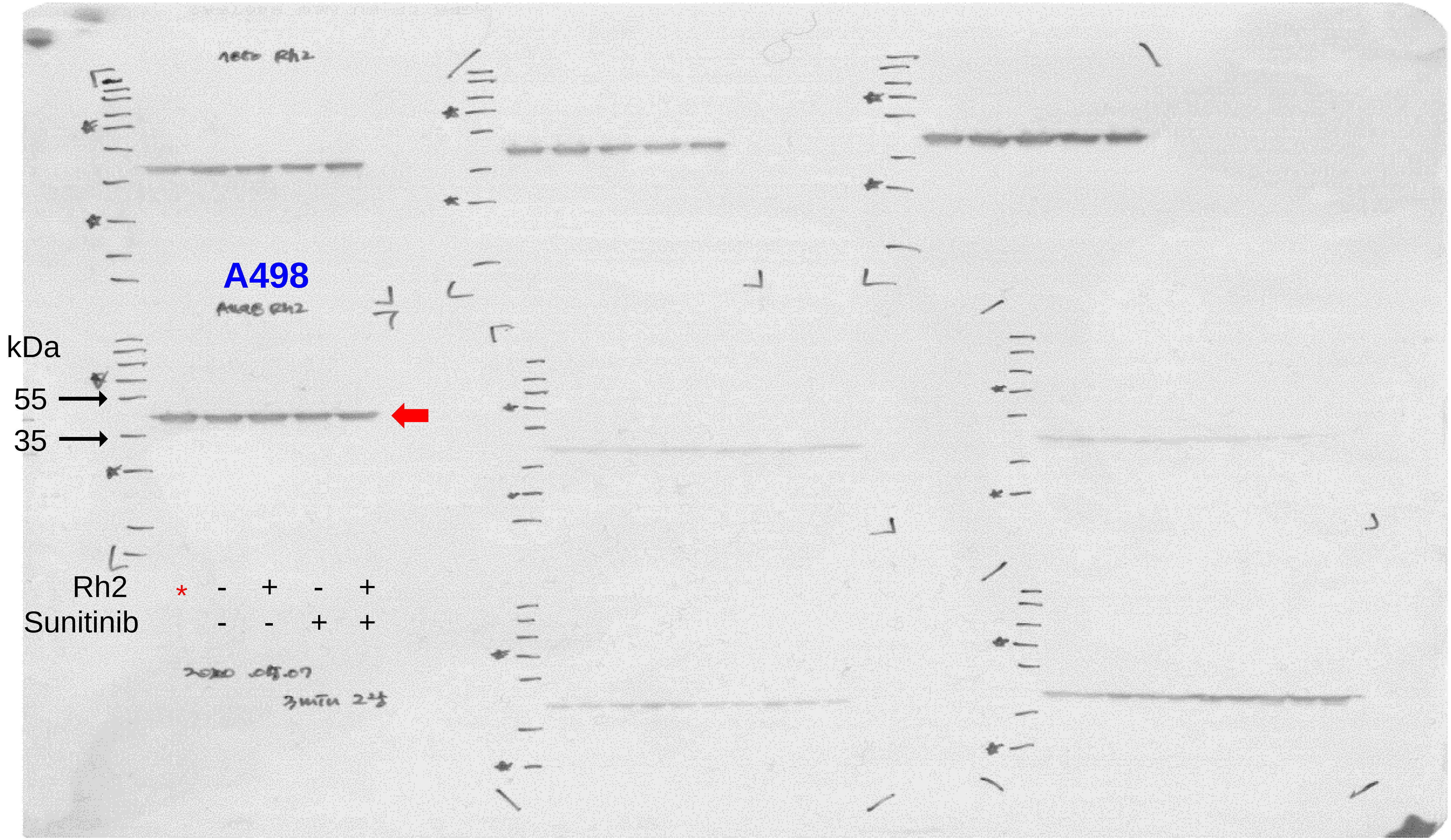

Supplementary Figure 2.
P53 (53 kDa)

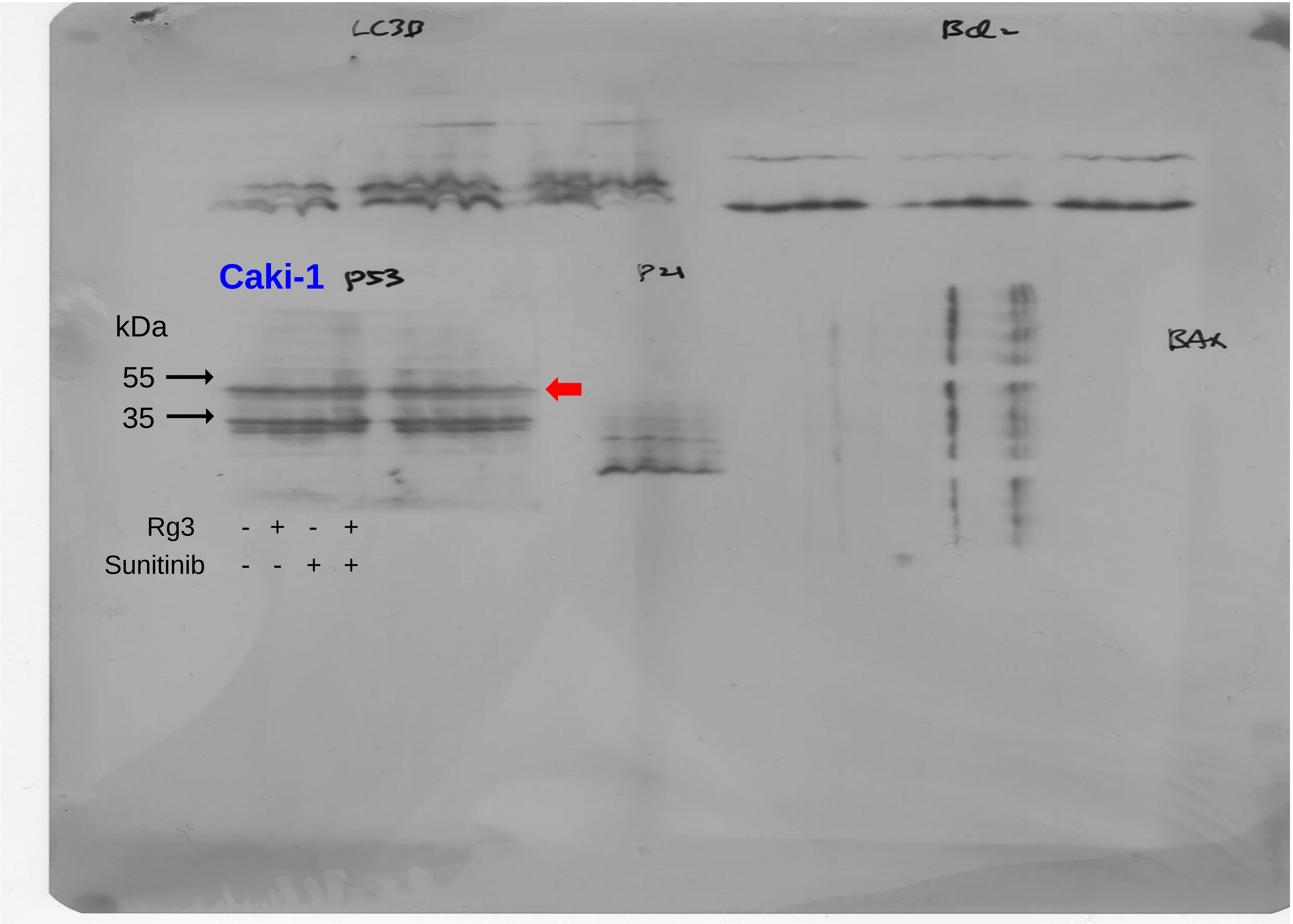

Supplementary Figure 2.
P53 (53 kDa)

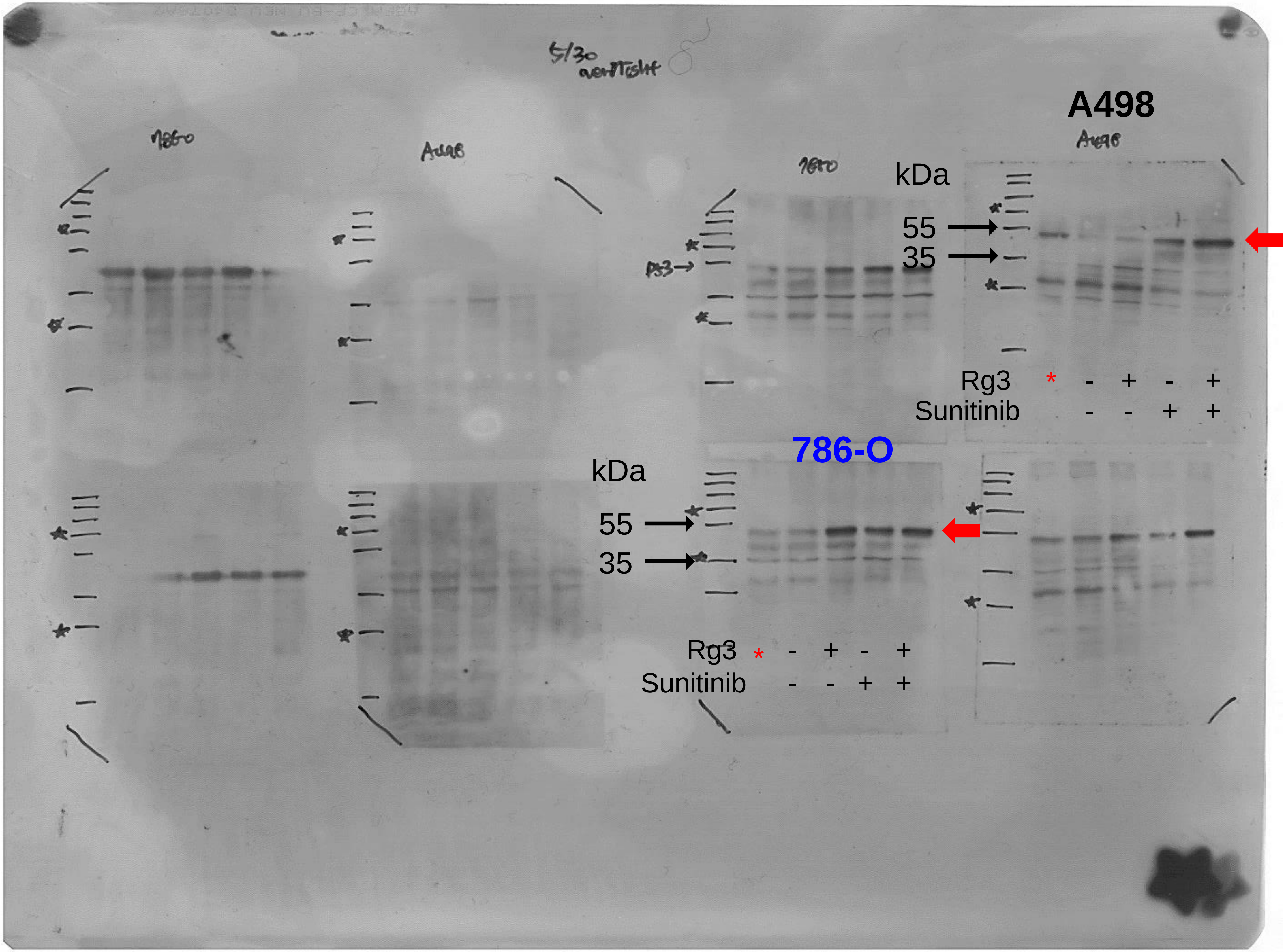

Supplementary Figure 2.

p-P53 (53 kDa)

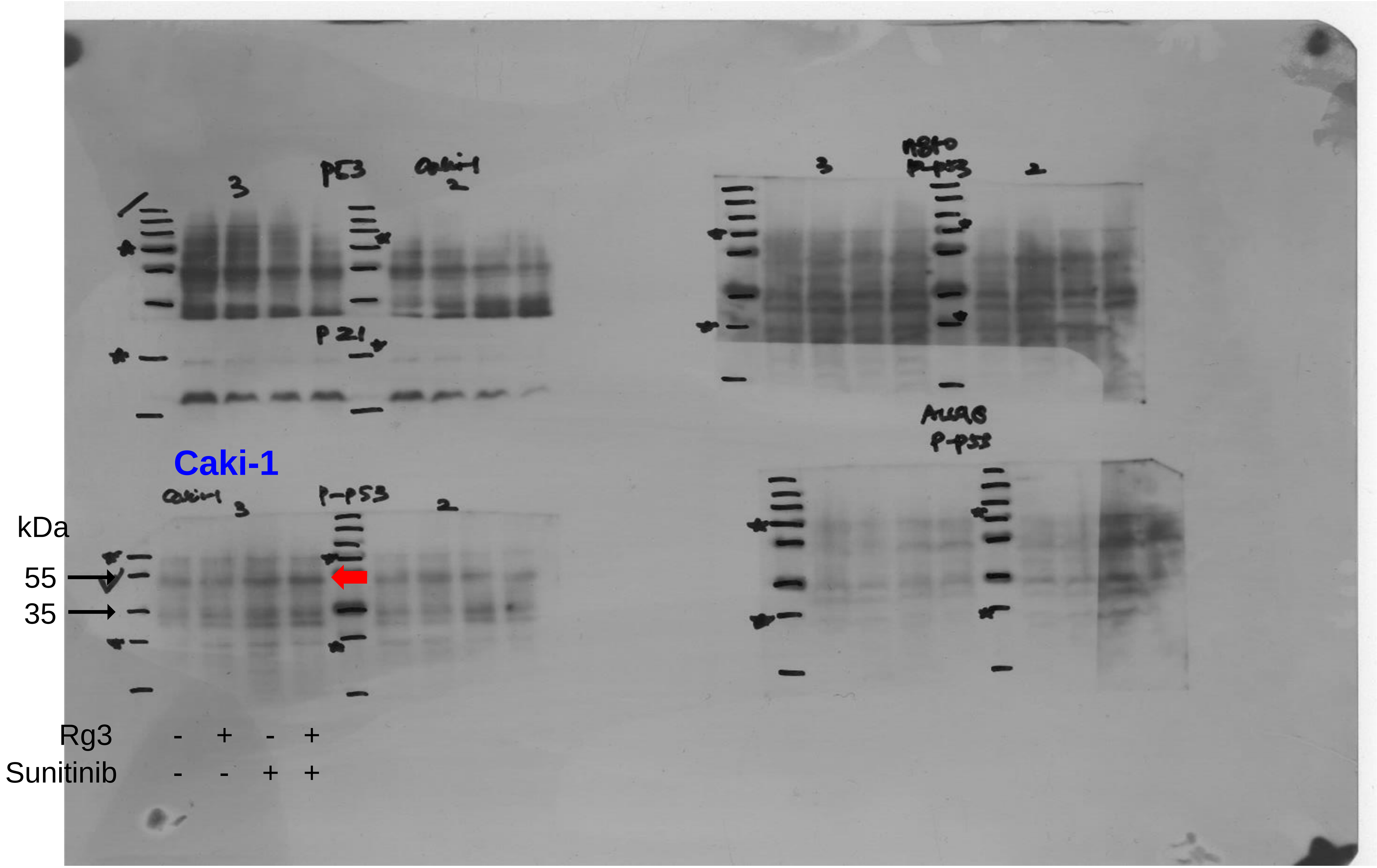

Supplementary Figure 2.

p-P53 (53 kDa)

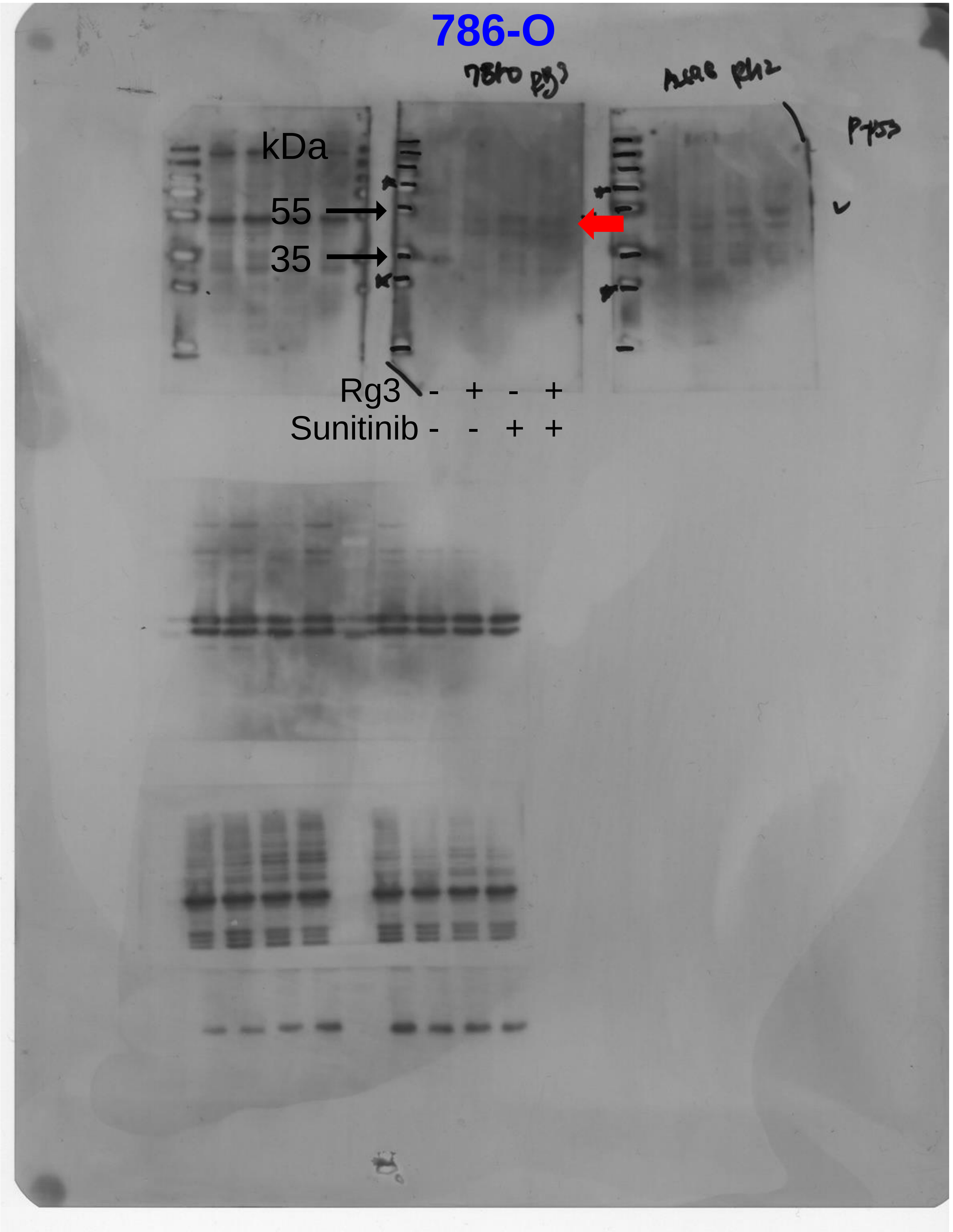

Supplementary Figure 2.

p-P53 (53 kDa)

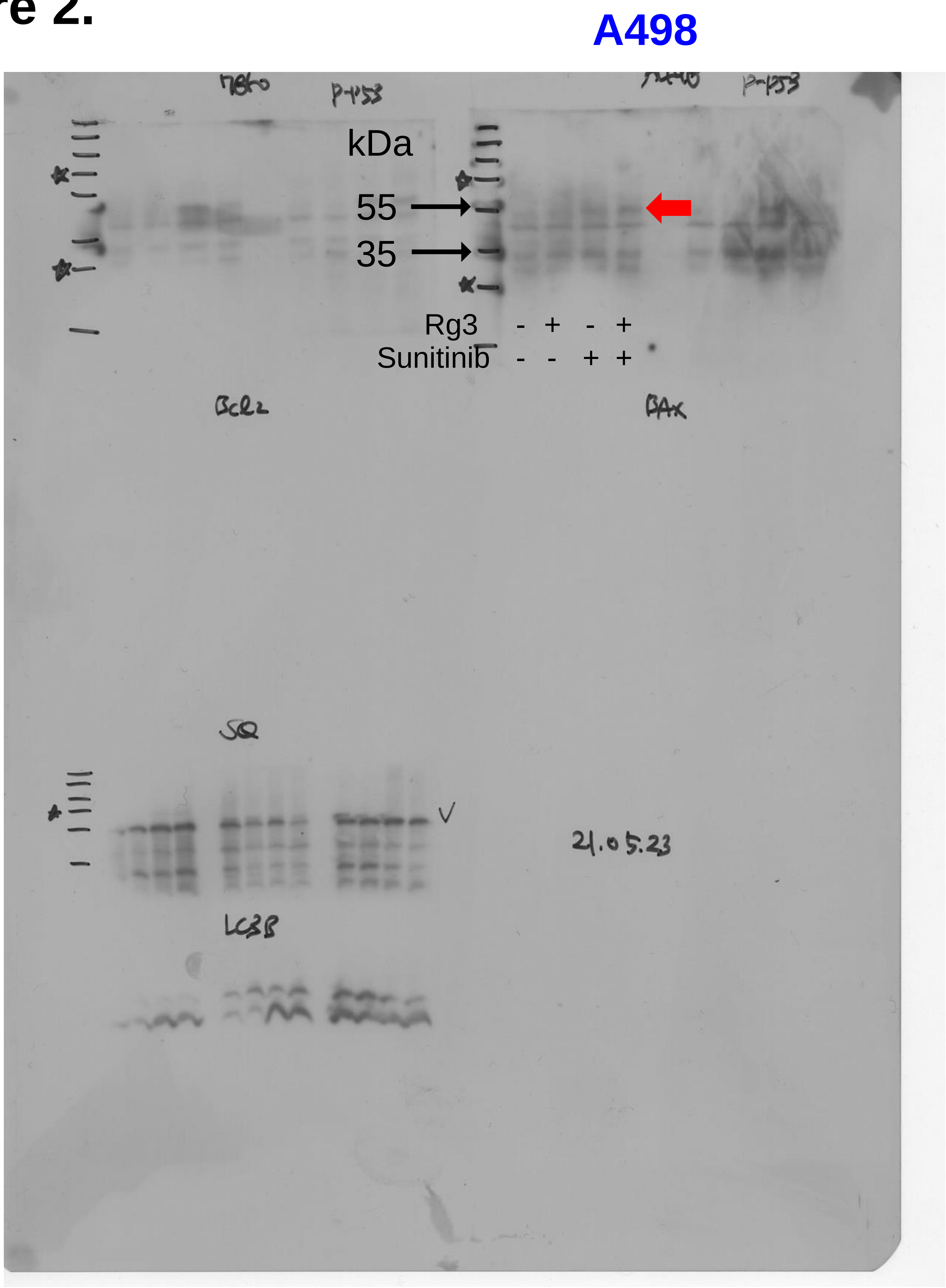

Supplementary Figure 2.

P21 (21 kDa)

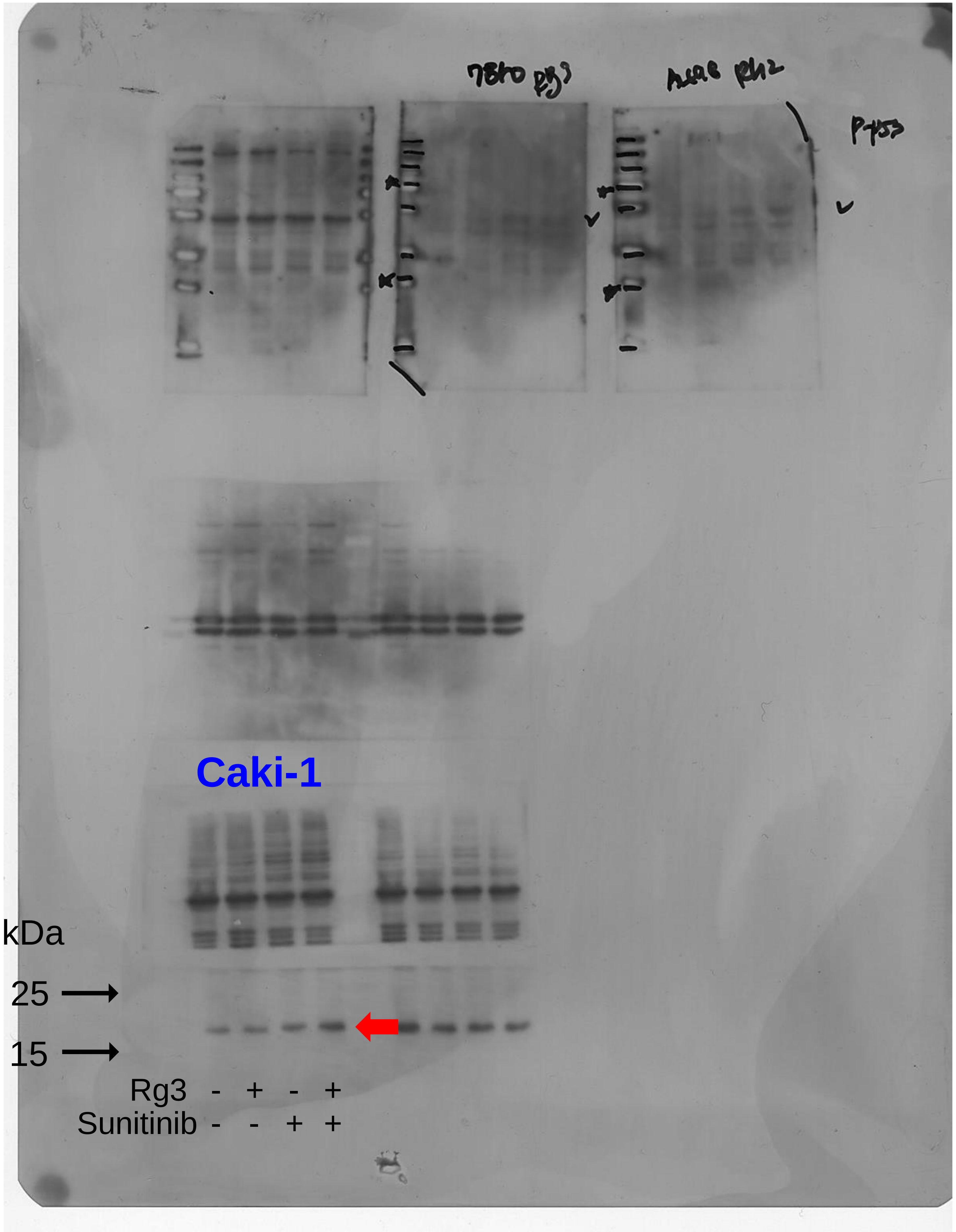

Supplementary Figure 2.

P21 (21 kDa)

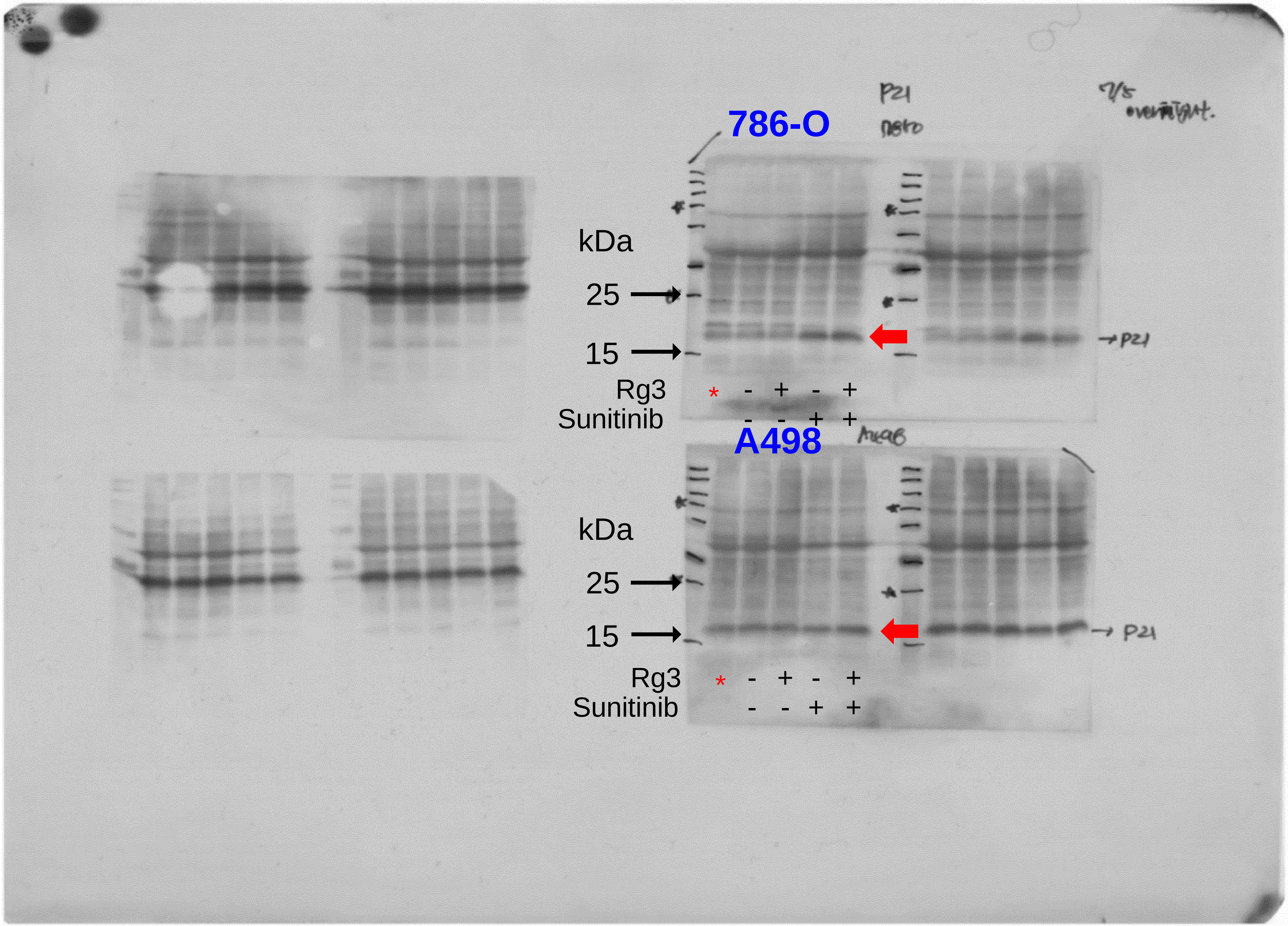

Supplementary Figure 2.

β actin (43 kDa)

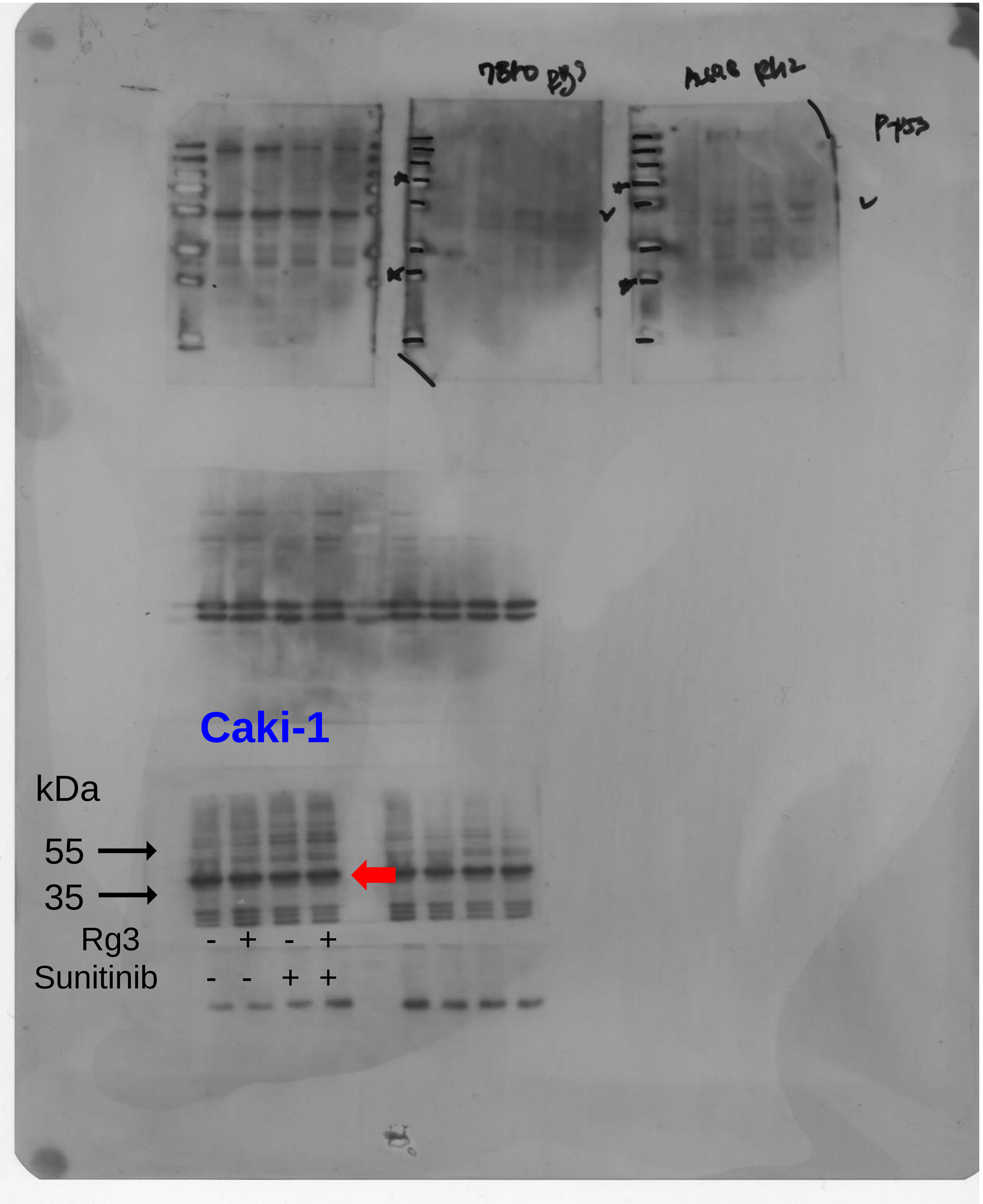

Supplementary Figure 2.

β actin (43 kDa)

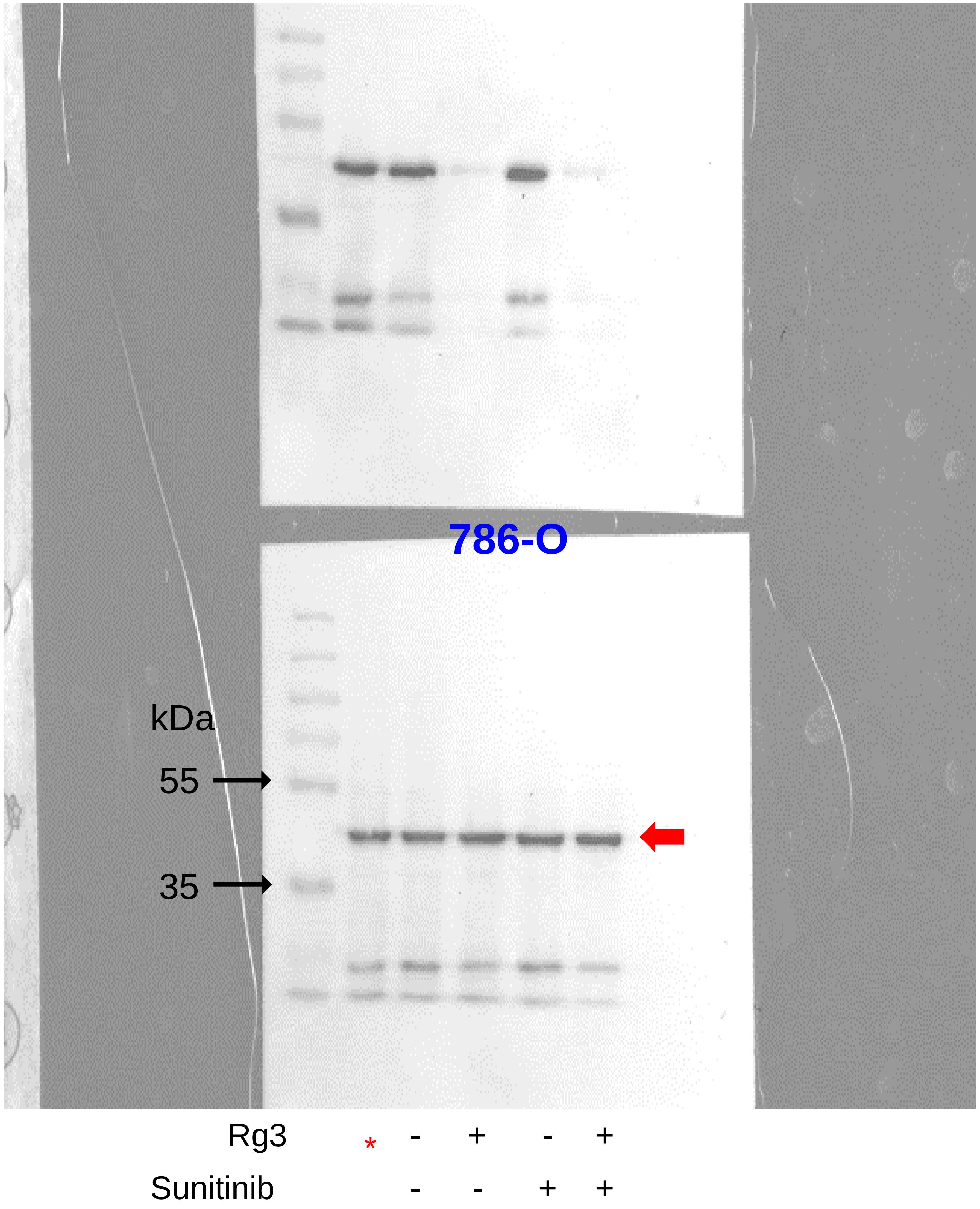

Supplementary Figure 2.

β actin (43 kDa)

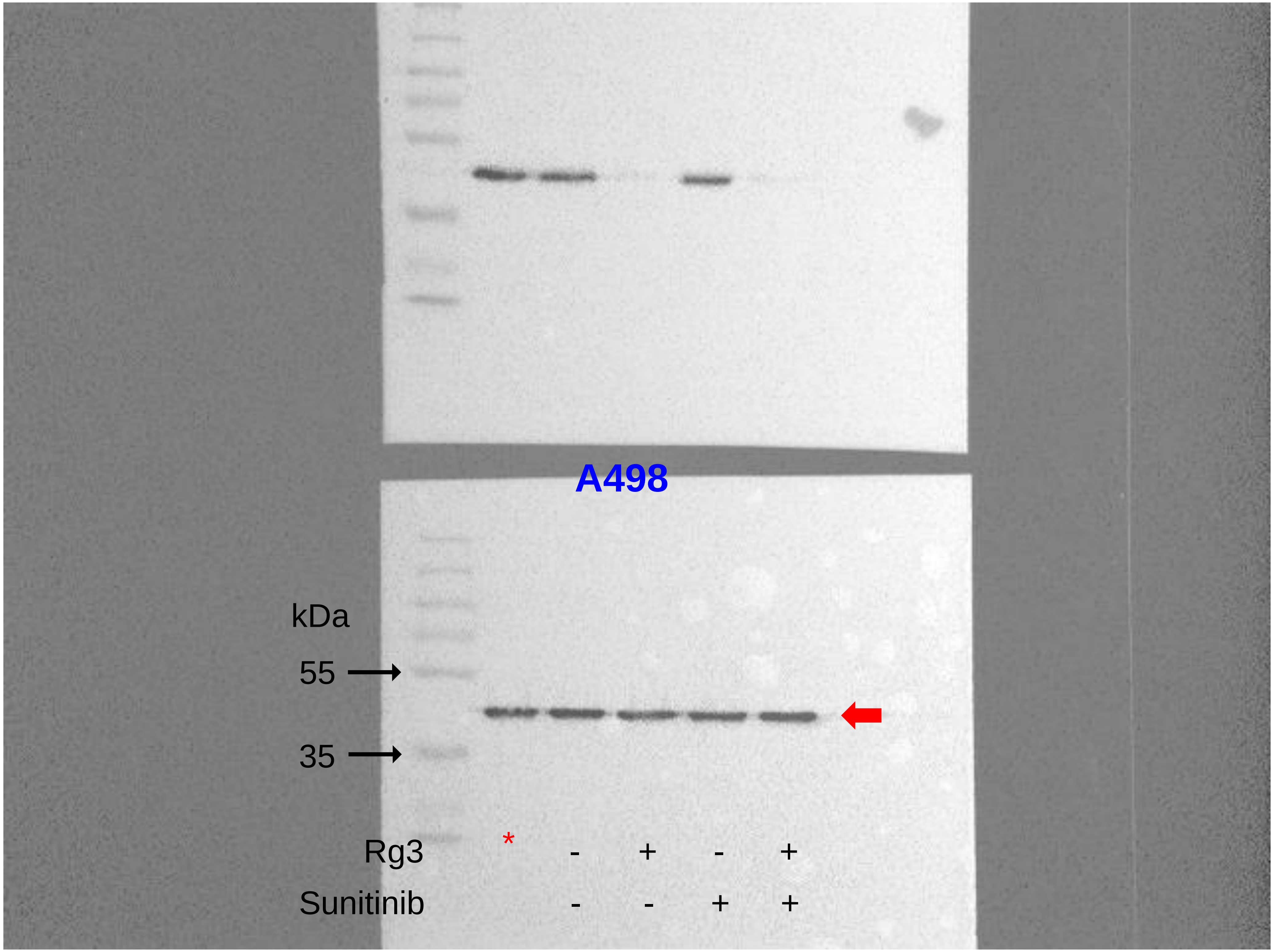

Figure 4.

P53 (53 kDa)

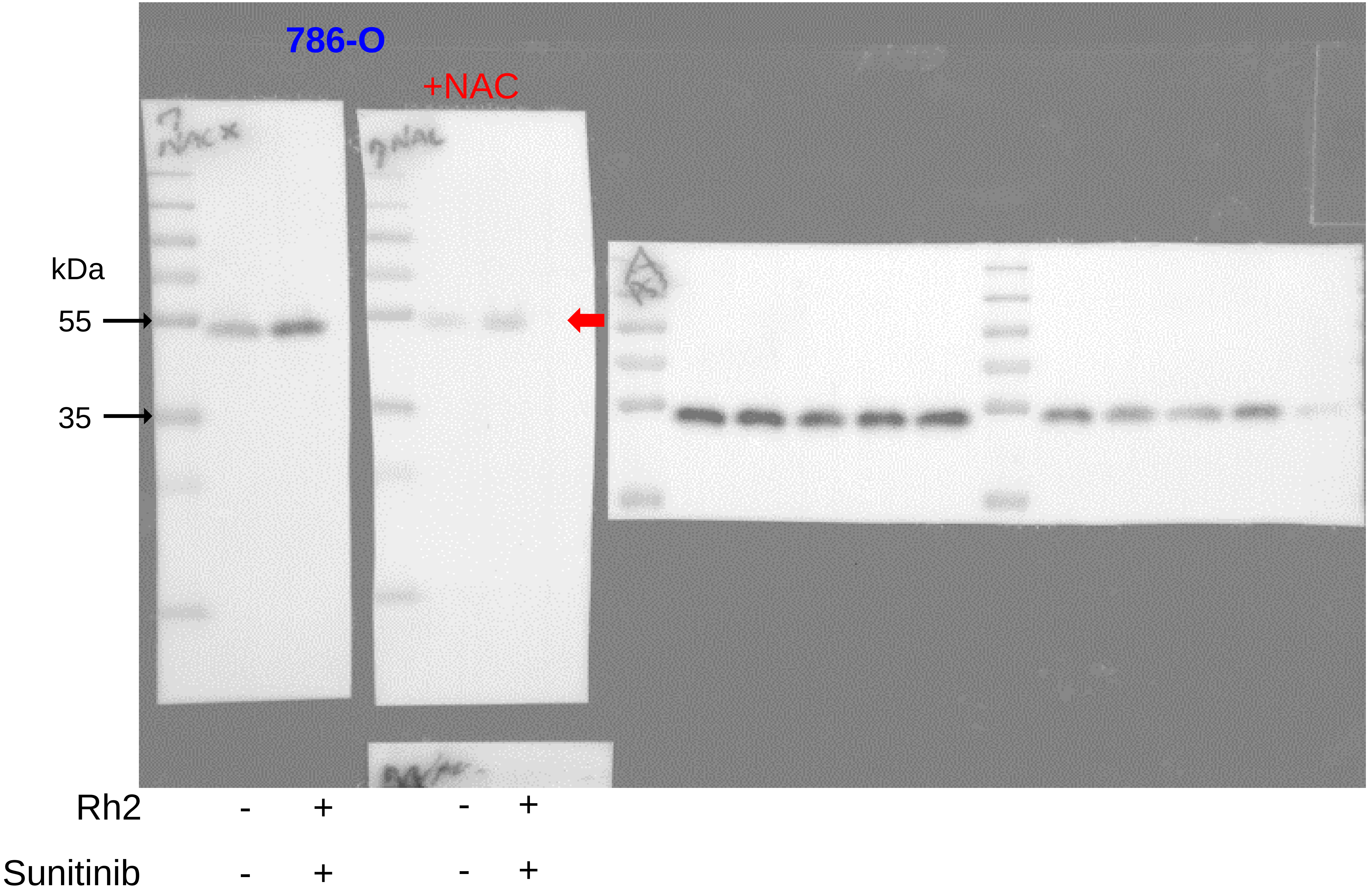

Figure 4.

p-P53 (53 kDa)

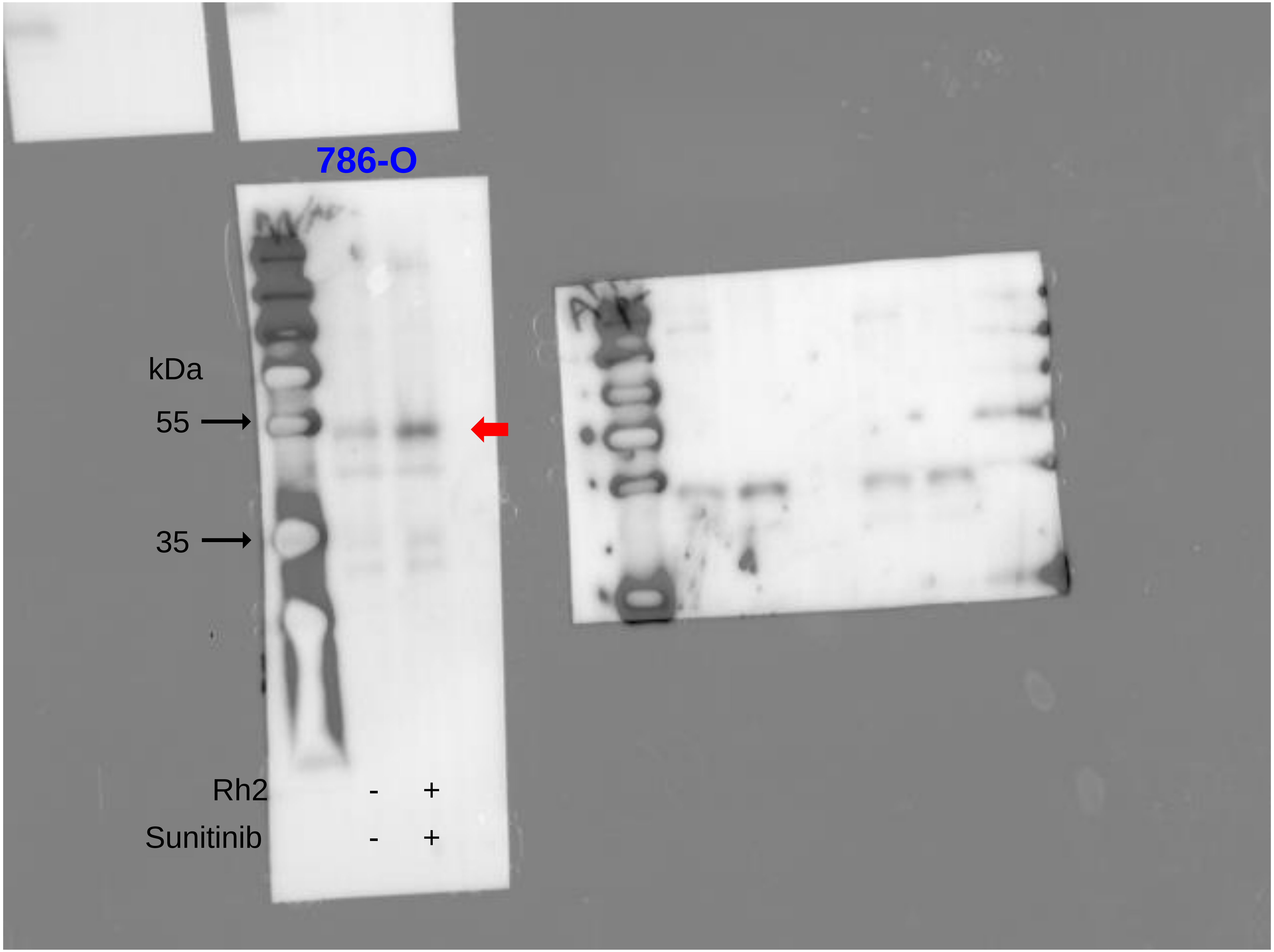

Figure 4.

p-P53 (53 kDa)

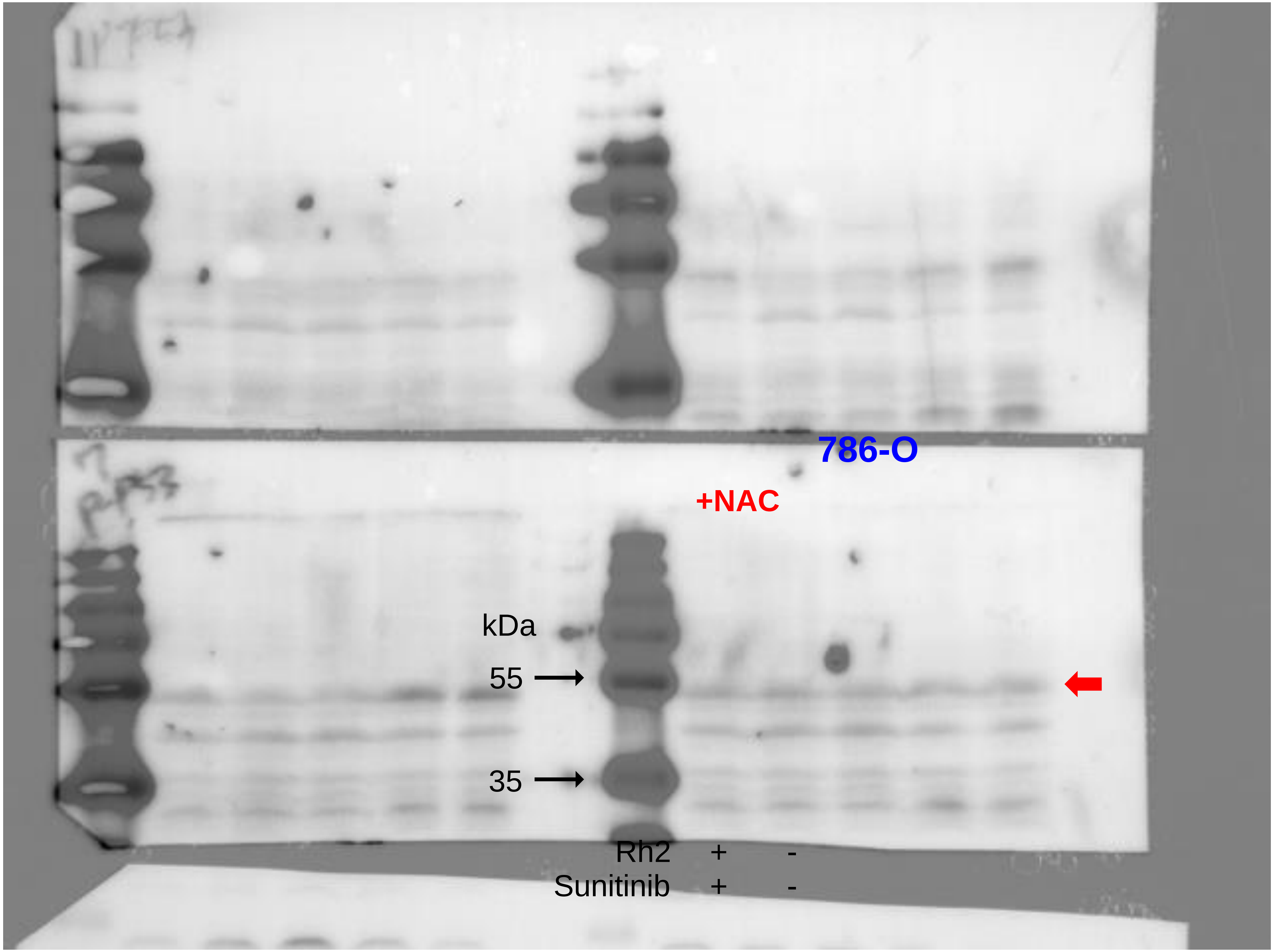

Figure 4.

P21 (21 kDa)

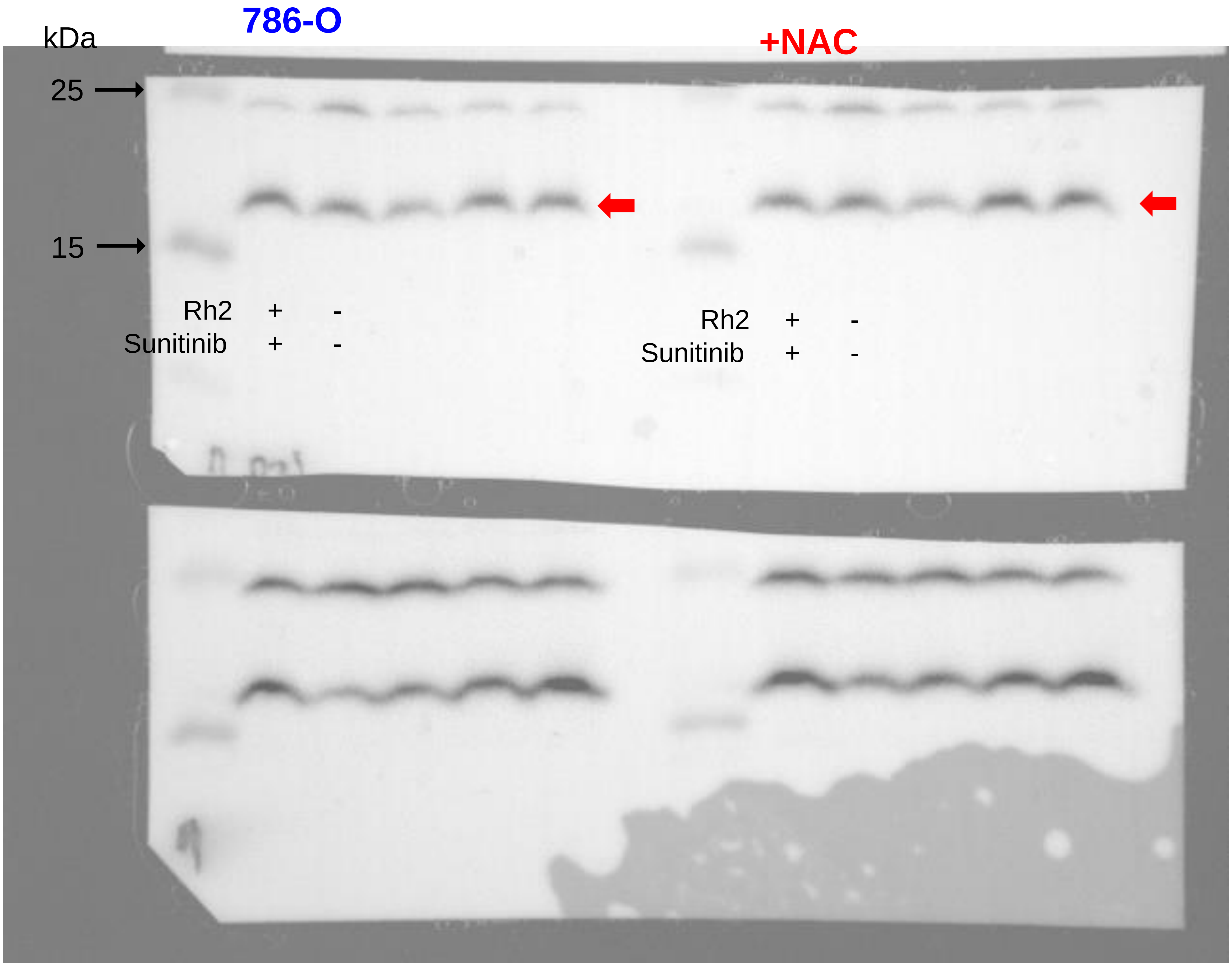

Figure 4.

β actin (43 kDa)

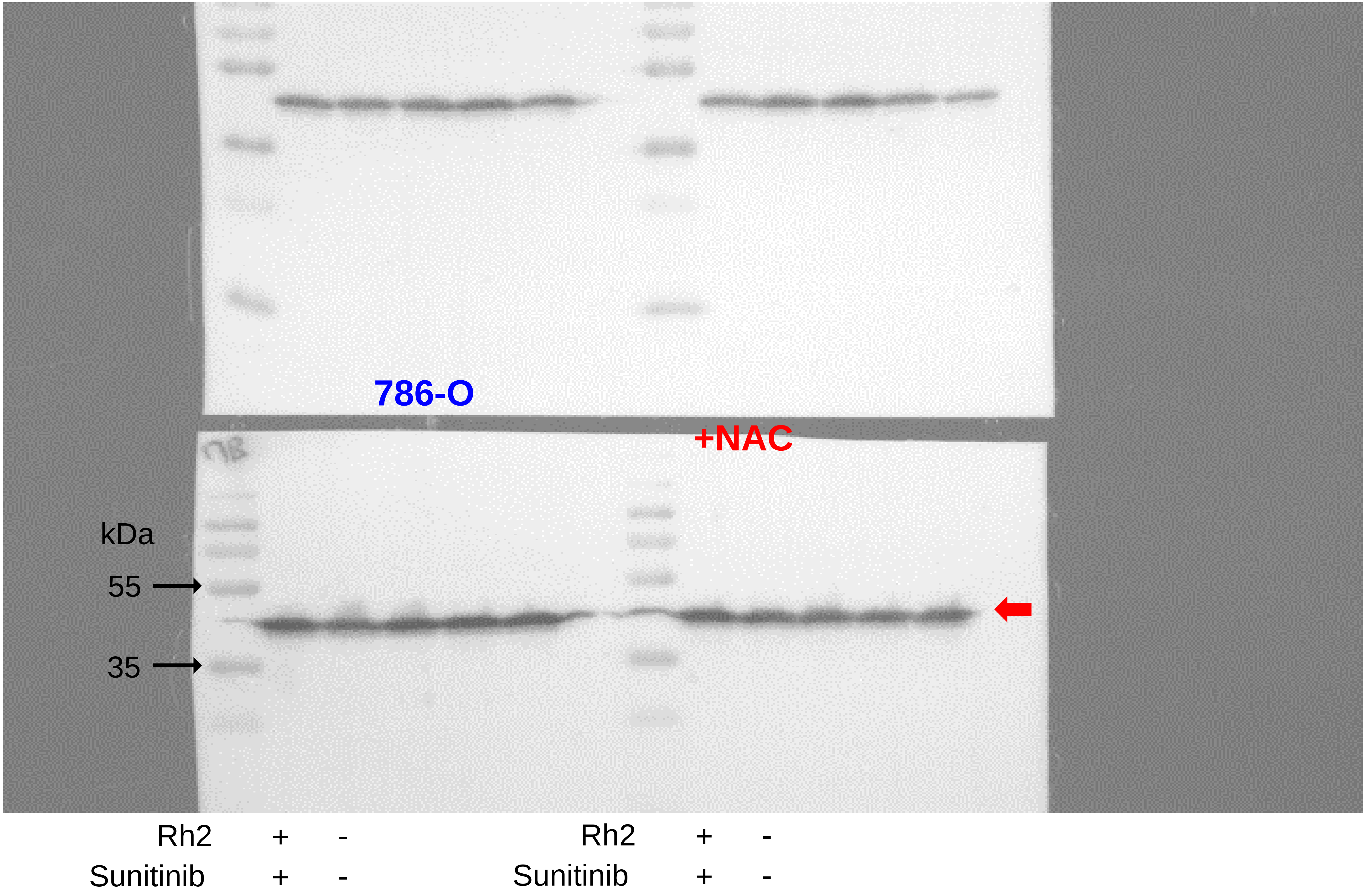

Supplementary Figure 4.
P53 (53 kDa)

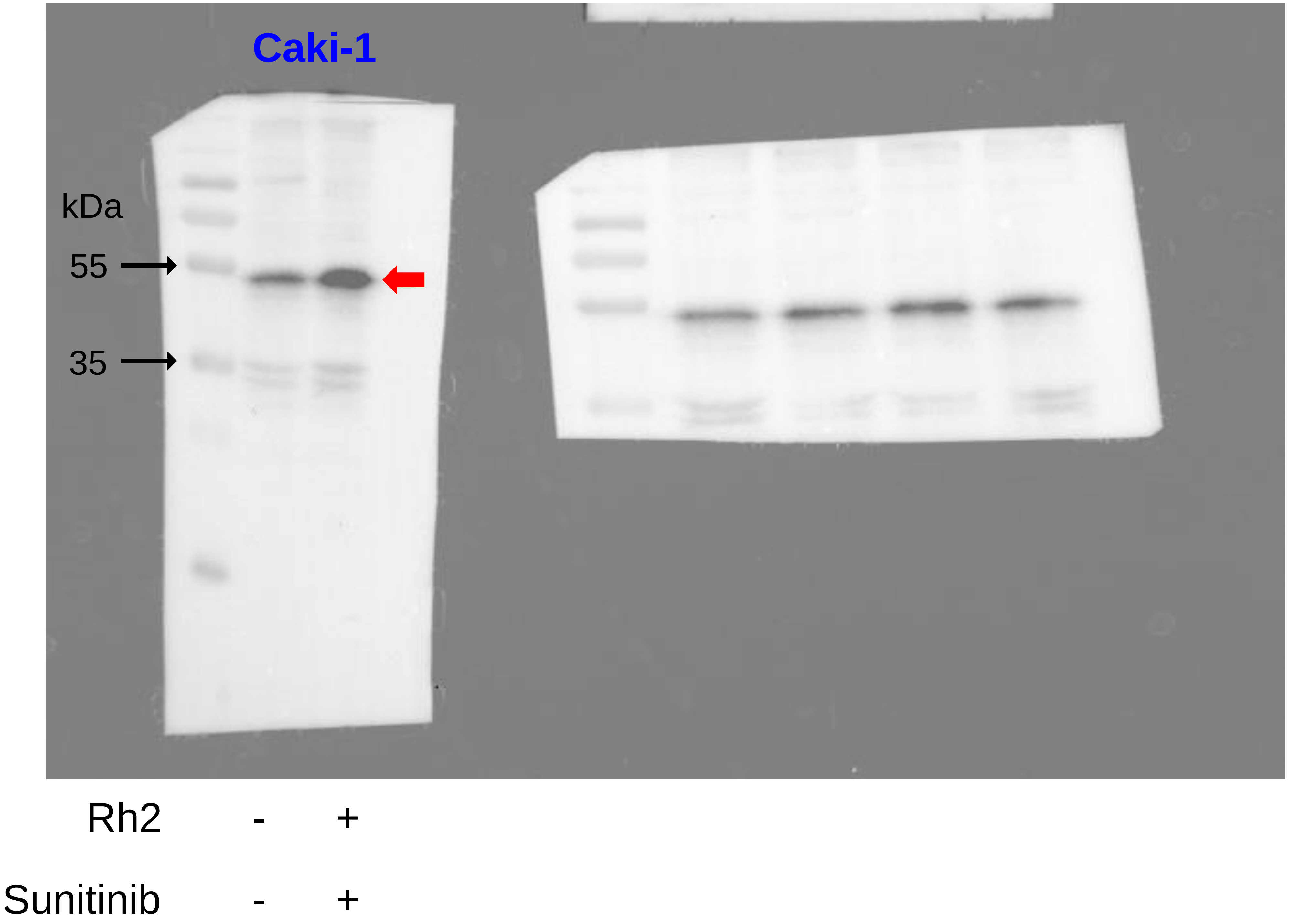

Supplementary Figure 4.
P53 (53 kDa)

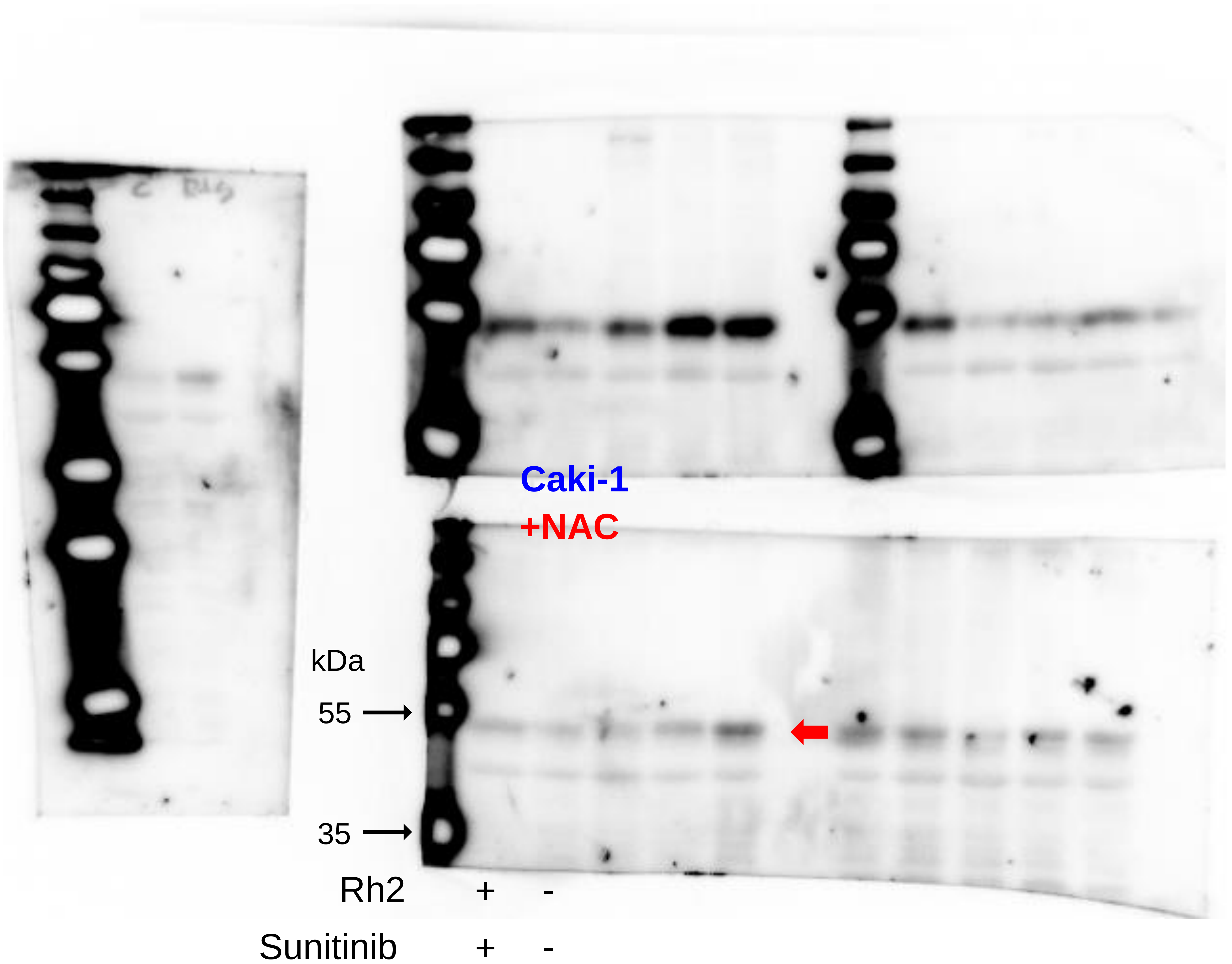

Supplementary Figure 4.
p-P53 (53 kDa)

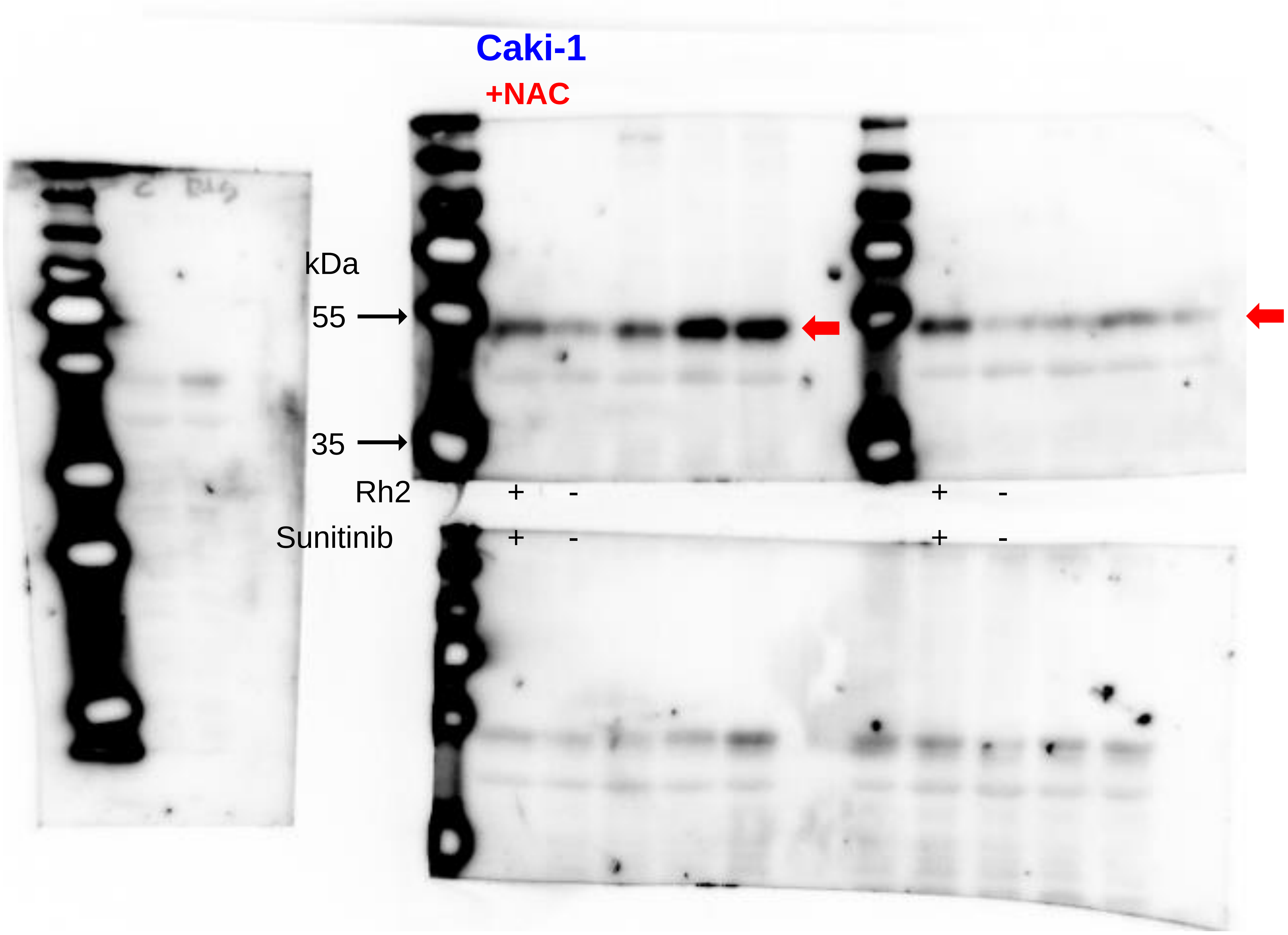

Supplementary Figure 4.
P21 (21 kDa)

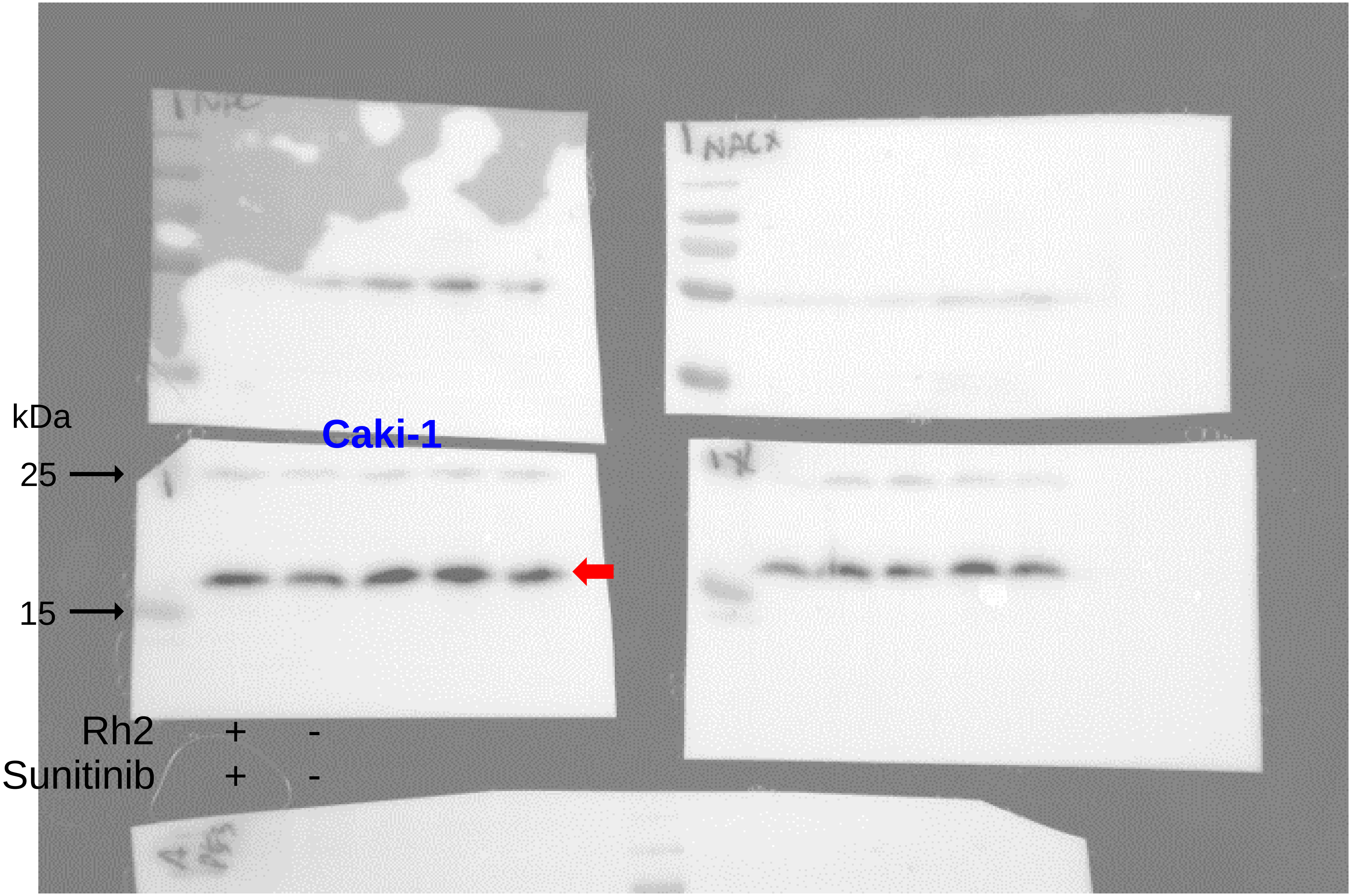

Supplementary Figure 4.
P21 (21 kDa)

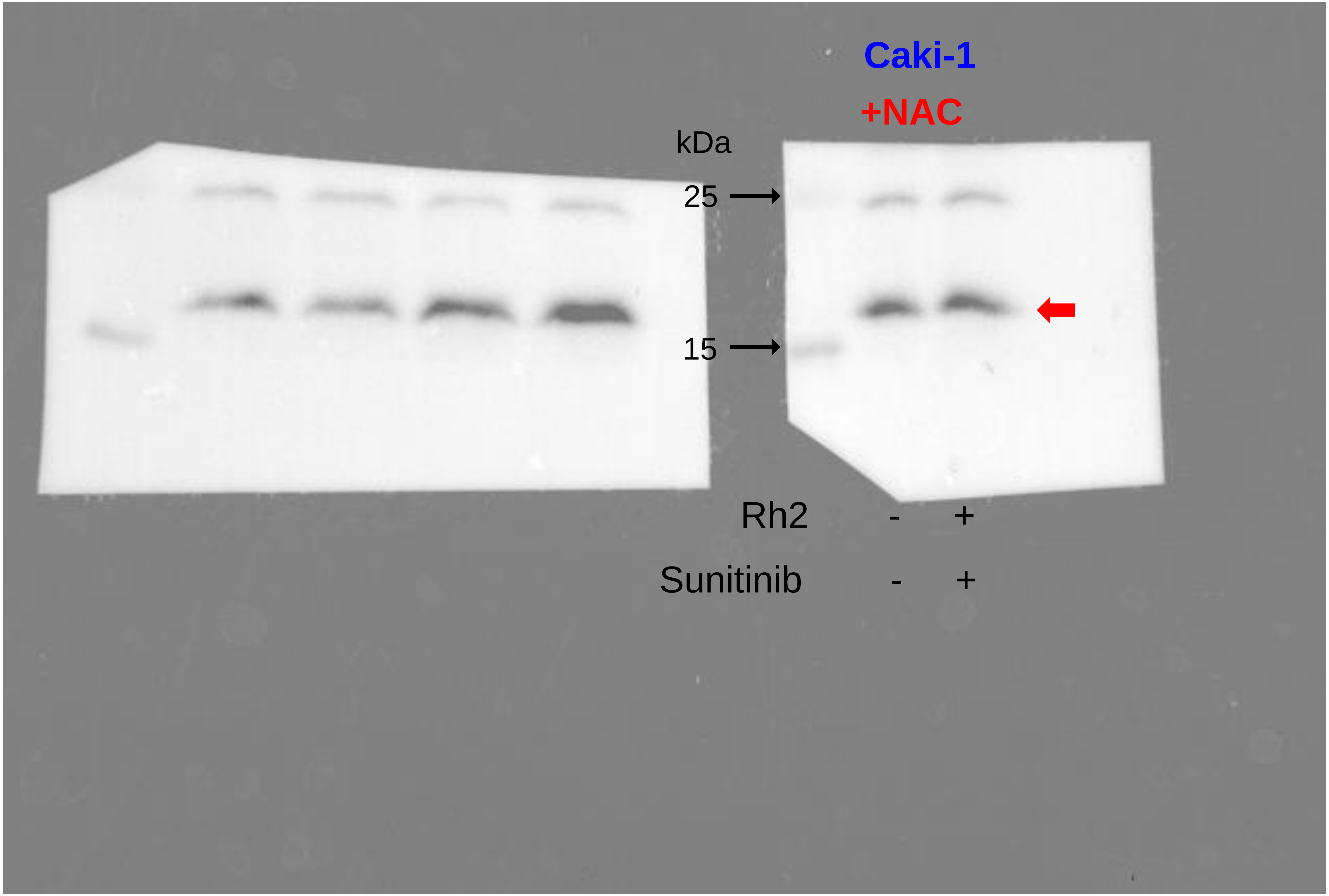

Supplementary Figure 4.
β actin (43 kDa)

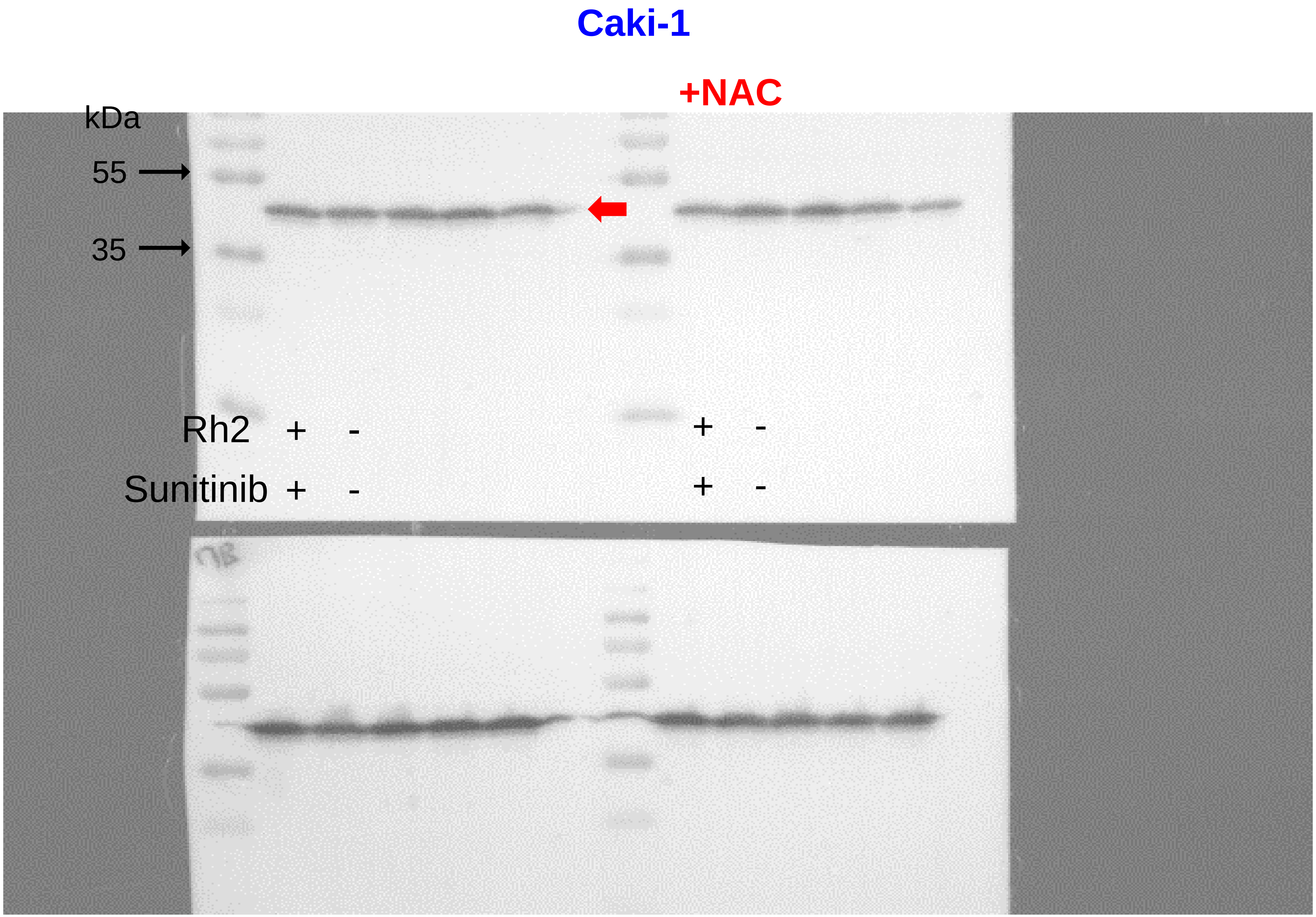

Supplementary Figure 4.
P53 (53 kDa)

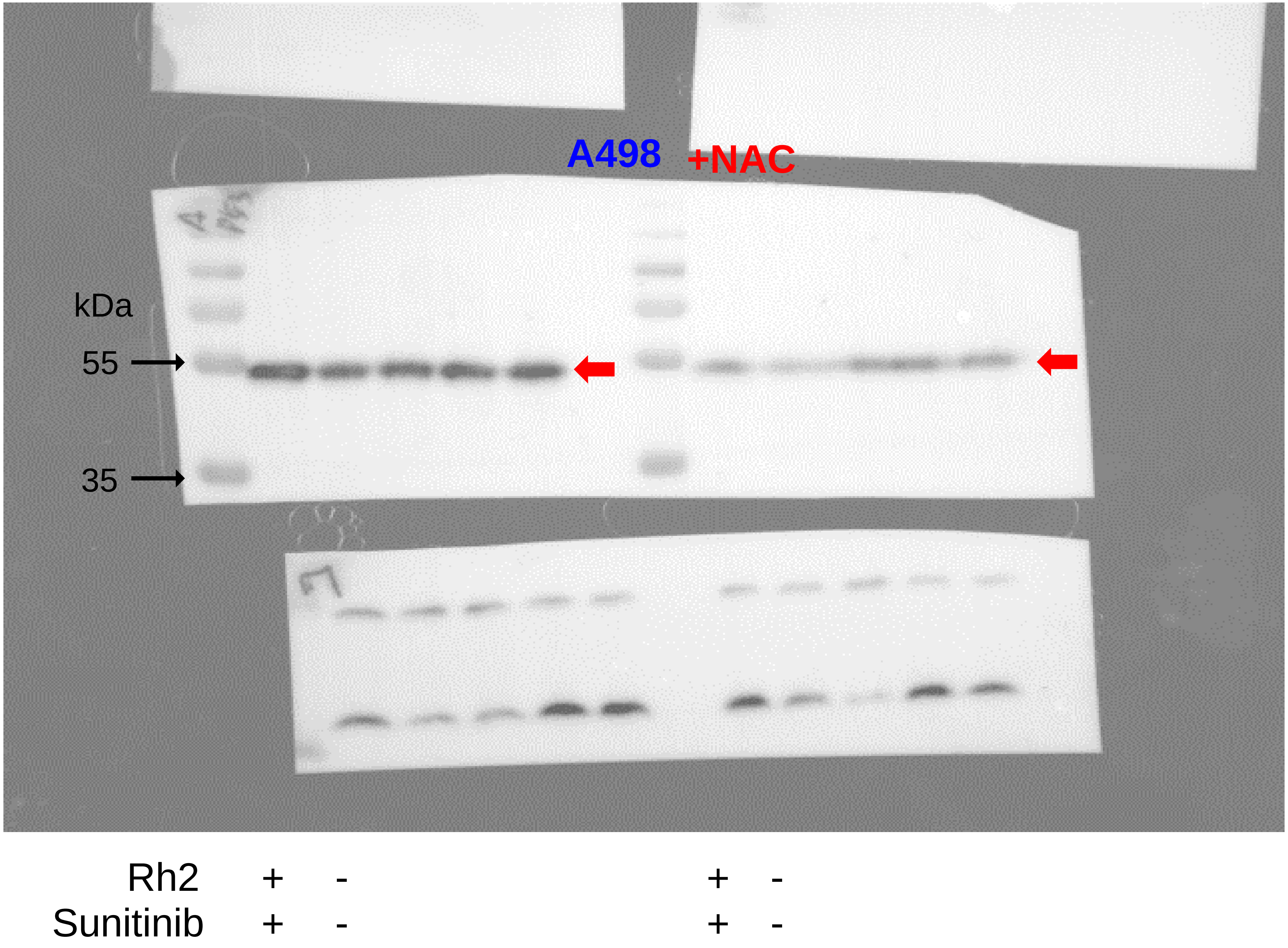

Supplementary Figure 4.
p-P53 (53 kDa)

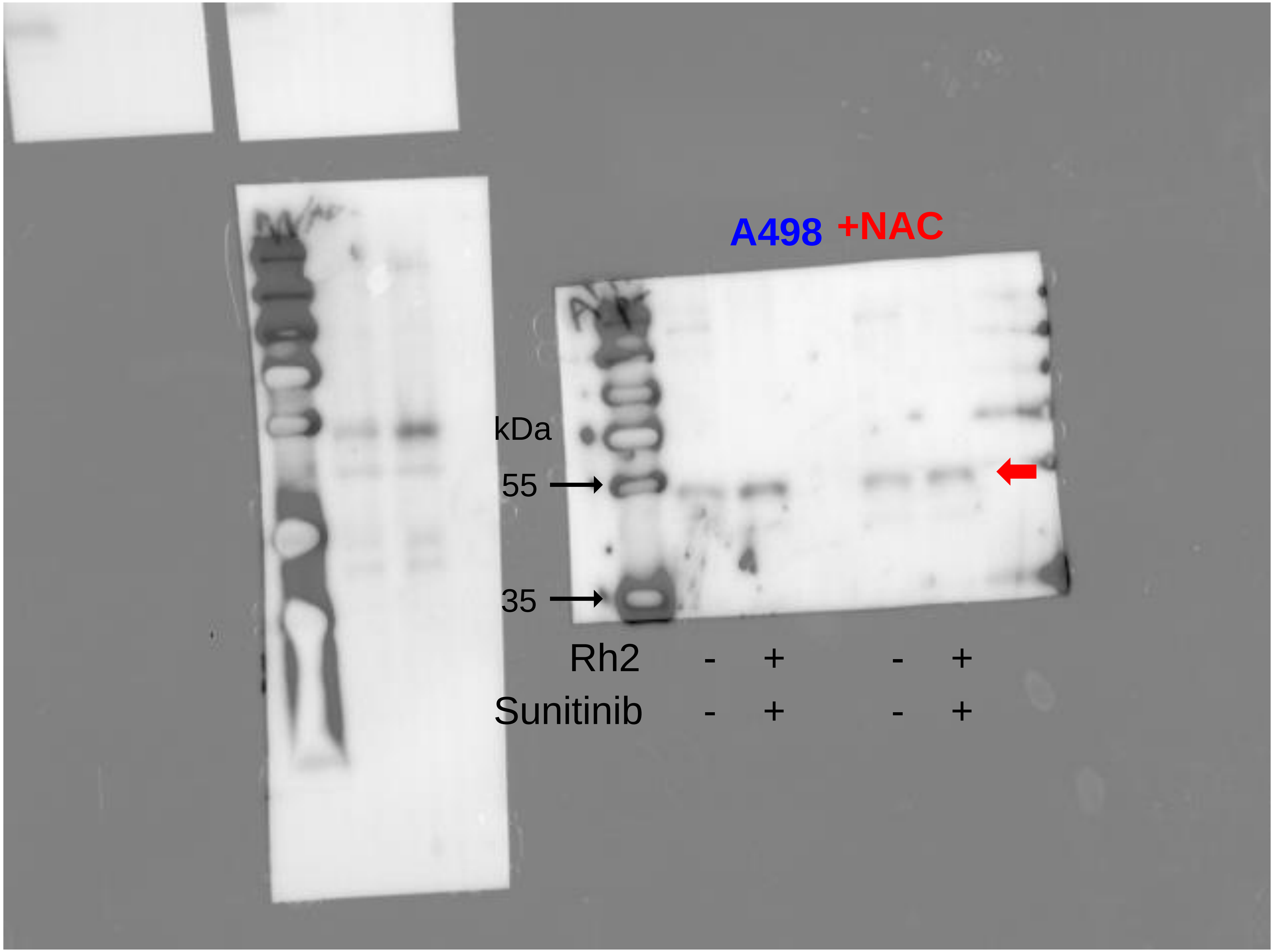

Supplementary Figure 4.
P21 (21 kDa)

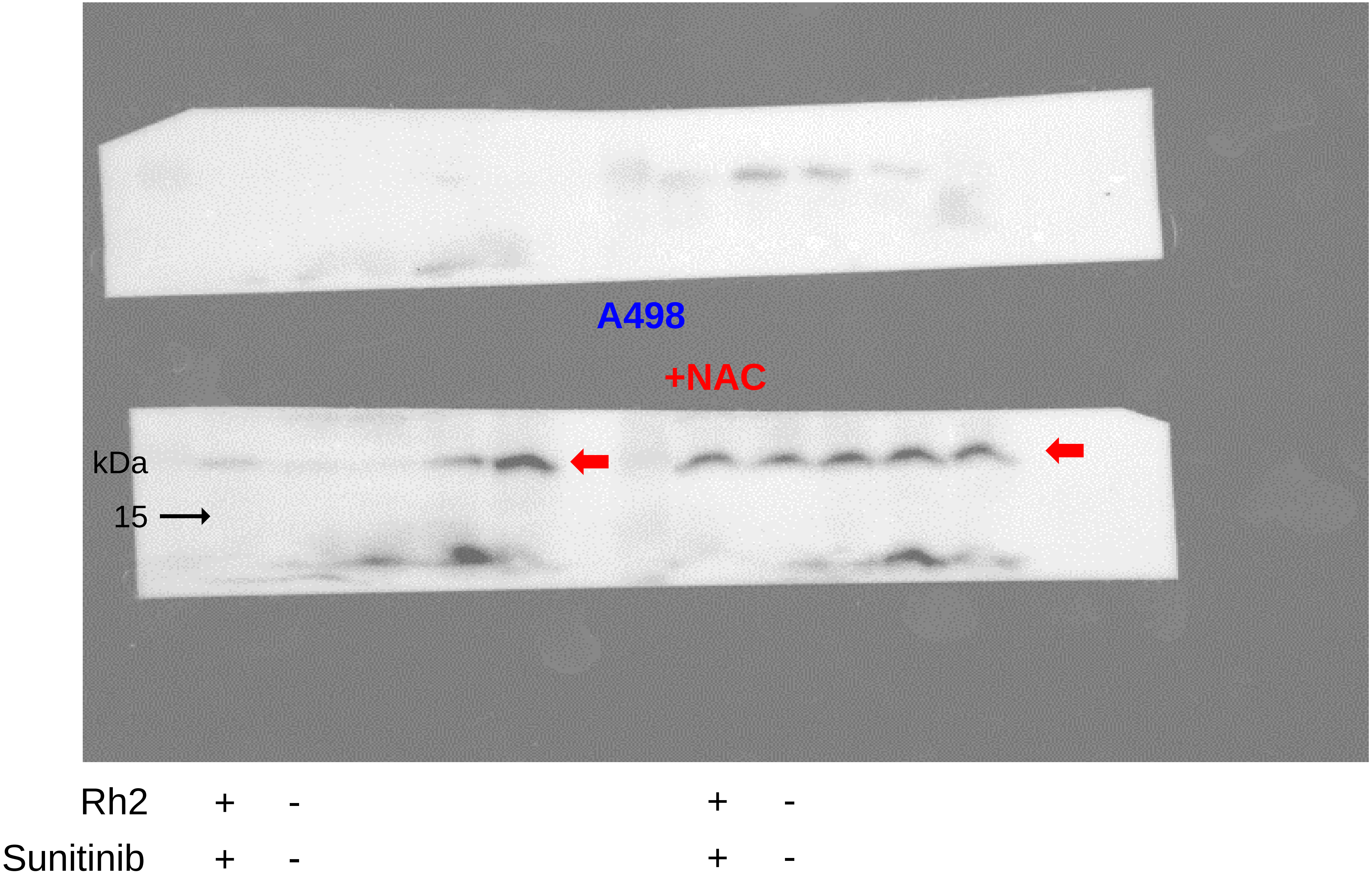

Supplementary Figure 4.
β actin (43 kDa)

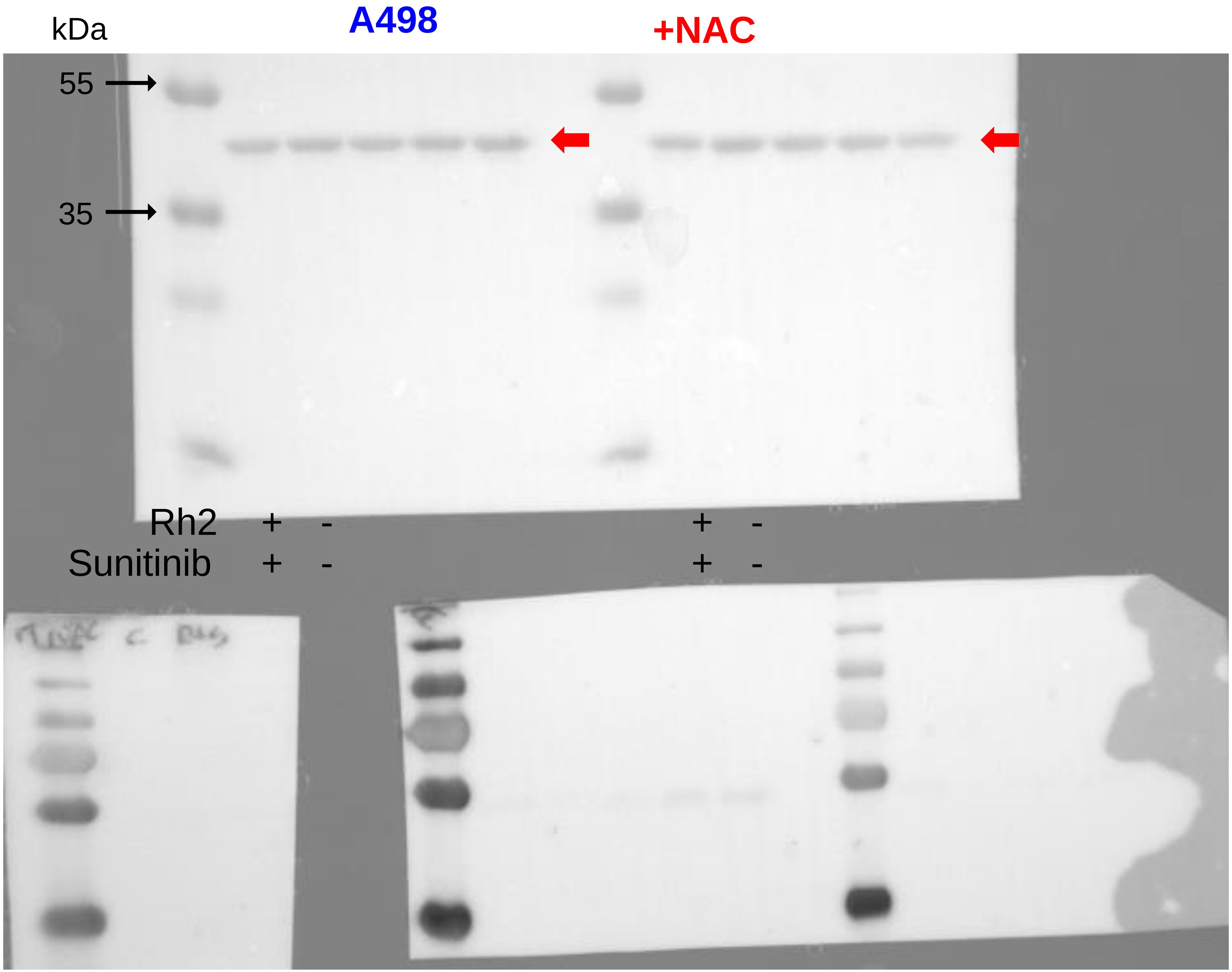

Supplement: Supplementary file 5 — Supplementary Information. [file 41598_2022_20075_MOESM5_ESM.pdf]
